# Gene Structural Specificity and Expression of *MADS-Box* Gene Family in *Camellia chekiangoleosa*

**DOI:** 10.3390/ijms24043434

**Published:** 2023-02-08

**Authors:** Pengyan Zhou, Yanshu Qu, Zhongwei Wang, Bin Huang, Qiang Wen, Yue Xin, Zhouxian Ni, Li’an Xu

**Affiliations:** 1 Co-Innovation Center for Sustainable Forestry in Southern China, Key Laboratory of Forest Genetics and Biotechnology Ministry of Education, Nanjing Forestry University, Nanjing 210037, China; 2 Jiangsu Key Laboratory for the Research and Utilization of Plant Resources, Institute of Botany, Jiangsu Province and Chinese Academy of Sciences, Nanjing 210014, China; 3 Jiangxi Provincial Key Laboratory of Camellia Germplasm Conservation and Utilization, Jiangxi Academy of Forestry, Nanchang 330047, China

**Keywords:** MADS-box, *Camellia chekiangoleosa*, floral organ, seed development, expression patterns

## Abstract

*MADS-box* genes encode transcription factors that affect plant growth and development. *Camellia chekiangoleosa* is an oil tree species with ornamental value, but there have been few molecular biological studies on the developmental regulation of this species. To explore their possible role in *C. chekiangoleosa* and lay a foundation for subsequent research, 89 *MADS-box* genes were identified across the whole genome of *C. chekiangoleosa* for the first time. These genes were present on all the chromosomes and were found to have expanded by tandem duplication and fragment duplication. Based on the results of a phylogenetic analysis, the 89 *MADS-box* genes could be divided into either type I (38) or type II (51). Both the number and proportion of the type II genes were significantly greater than those of *Camellia sinensis* and *Arabidopsis thaliana*, indicating that *C. chekiangoleosa* type II genes experienced a higher duplication rate or a lower loss rate. The results of both a sequence alignment and a conserved motif analysis suggest that the type II genes are more conserved, meaning that they may have originated and differentiated earlier than the type I genes did. At the same time, the presence of extra-long amino acid sequences may be an important feature of *C. chekiangoleosa*. Gene structure analysis revealed the number of introns of *MADS-box* genes: twenty-one type I genes had no introns, and 13 type I genes contained only 1~2 introns. The type II genes have far more introns and longer introns than the type I genes do. Some MIKC^C^ genes have super large introns (≥15 kb), which are rare in other species. The super large introns of these MIKC^C^ genes may indicate richer gene expression. Moreover, the results of a qPCR expression analysis of the roots, flowers, leaves and seeds of *C. chekiangoleosa* showed that the *MADS-box* genes were expressed in all those tissues. Overall, compared with that of the type I genes, the expression of the type II genes was significantly higher. The *CchMADS31* and *CchMADS58* genes (type II) were highly expressed specifically in the flowers, which may in turn regulate the size of the flower meristem and petals. *CchMADS55* was expressed specifically in the seeds, which might affect seed development. This study provides additional information for the functional characterization of the *MADS-box* gene family and lays an important foundation for in-depth study of related genes, such as those involved in the development of the reproductive organs of *C. chekiangoleosa*.

## 1. Introduction

Oil tea is one of the four major types of oil trees in the world and generally refers to any one of several *Camellia* plant species that are in the Theaceae family and have a high seed oil content. Oil tea species are the most important woody oil plant species in China. *Camellia chekiangoleosa* Hu, one of the main cultivars in China, has a short fruit ripening period, and its seeds have a high oil content. *C. chekiangoleosa* is rich in a variety of high-priced unsaturated fatty acids, which are collectively known as “oriental olive oil”. *C. chekiangoleosa* is also an industrial raw material for soaps, lubricants, and pharmaceuticals. In addition, it is a famous garden ornamental plant species because of its winter and spring flowering, long flowering period, and large and colorful flowers [1]. However, early research of *C. chekiangoleosa* mainly focused on germplasm resource conservation, genetic diversity analysis, molecular marker-assisted breeding, and other aspects [2,3,4]. There are few reports on the molecular biology of this species, such as that which drives developmental regulation.

Transcription factors (TFs) play a variety of roles throughout the life cycle of higher plants [5]. TFs bind to cis-acting regulatory sequences and are involved in plant growth, development, morphogenesis, and stress responses [6]. The *MADS-box* gene family is a large family whose members encode TFs, and four major *MADS-box* genes encoding TFs have been discovered: MCM1 of *Saccharomyces cerevisiae* [7], AG of *Arabidopsis thaliana* [8], DEF of *Antirrhinum majus* [9], and SRF of humans [7]. Based on their structure and evolutionary developmental state, *MADS-box* genes can be divided into two subtypes: SRF-like (type I) and MEF2-like (type II) [10]. The type I genes can be further divided into three subfamilies: Mα, Mβ, and Mγ. However, few type I genes have biological functions. Only a small number of type I genes, which affect the reproductive development of *A. thaliana*, have been reported. In contrast, the type II genes have been studied more in depth and extensively. Type II genes, which are also known as the MIKC type due to their common structure, can be further divided into two subtypes (MIKC^C^ and MIKC*) based on different structural features [11]. The MIKCC subfamily can be divided into at least 14 different subclasses, such as STK, PI, AGL12, and SEP [12,13].

Previous studies have shown that MIKC^C^-type genes play an important role in plant floral organ development [14,15]. Molecular and genetic analysis subdivided these genes into five different classes (A, B, C, D, and E), to specify the identity of sepals (A), petals (A + B + E), stamens (B + C + E), carpels (C + E), and ovules (D) [12,15,16]. The genes belonging to the above five functional categories in *A*. *thaliana* include *APETALA1* (*AP1*) and *AP2* in class A, *PISTILATA* (*PI*) and *APETALA3* (*AP3*) in class B, *AGAMOUS* (*AG*) in class C, *SEEDSTICK*/*AGAMOUS-LIKE 1* (*STK*/*AGL11*) and *SHATTERPROOF* (*SHP*) in class D, and *SEPALLATA* (*SEP1*, *SEP2*, *SEP3*, SEP4) genes in class E [17,18,19,20,21].

The expression of type II genes in organs such as fruits and seeds is also common. Type II genes play an important role in seed development [22,23]. Type E (*SEP*) genes not only play a role in the development of flower organs and the maintenance of meristem characteristics in tomato (*TM29*) and strawberry (*FaMADS9*) but also regulate the development and maturation of their fruits [24,25]. The type D (*AGL11*/*STK*) gene *STK* promotes the association between seeds and fruits in *A. thaliana* [21]. After fruit ripening has occurred, the type A gene *AP1* and the type D genes *SHP1* and *SHP2* together regulate valve separation of fruits, allowing seed dispersal [26].

Genome-wide identification analysis and functional characterization of the *MADS-box* gene family have been performed on many organisms [27,28]. However, there have been no reports of these on *C. chekiangoleosa*. In this study, we analyzed the whole-genome data of *C. chekiangoleosa*. With the help of a bioinformatics platform and real-time quantitative techniques, whole-genome identification and analysis of the *MADS-box* gene family of *C. chekiangoleosa* were carried out. Aiming to provide a basis for the reproductive organ development of *C. chekiangoleosa* at the genetic level, we also measured gene expression.

## 2. Results

### 2.1. Identification and Analysis of Physicochemical Properties of MADS-Box Proteins

According to the results of a BLAST alignment and the hidden Markov model (HMM), 111 *MADS-box* genes were preliminarily screened from the whole genome of *C. chekiangoleosa*. Based on the *MADS-box* model of *A. thaliana*, a total of 89 domain-intact MADS-box proteins were obtained. The protein sequence is shown in File S1 of the Supplementary Materials. They all had a conserved MADS domain at the N-terminus, and this domain consisted of approximately 59 amino acid sequences. Multiple sequence alignments of the MADS domains of the 89 proteins and sequence icons (Figure S1) revealed four highly conserved amino acids (aa) (aa 21, 25, 32, and 39). According to the alignment results (Figure S2), we found that the difference between type I and type II MADS-box domains mainly involved differences in N-terminal aa; moreover, the domain of type II MADS-box proteins was more conserved.

The physicochemical properties of the MADS-box proteins of *C. chekiangoleosa* showed that the aa of the 89 MADS-box proteins ranged from 102 to 1528 aa (Table 1). The sequence length of most CchMADS proteins (61.8%) was 200~300 aa. In total, 24.7% of the proteins were between 100 and 200 aa in length, and the rest were greater than 300 aa. CchMADS76 was the longest (1528 aa). The predicted molecular weight was from 11.521 to 176.716 kDa, and the predicted isoelectric point ranged from 4.92 to 10.48. Subcellular localization predicted that all the proteins were localized in the nucleus.

### 2.2. Phylogenetic Analysis of CchMADS Proteins

**Figure 1:**
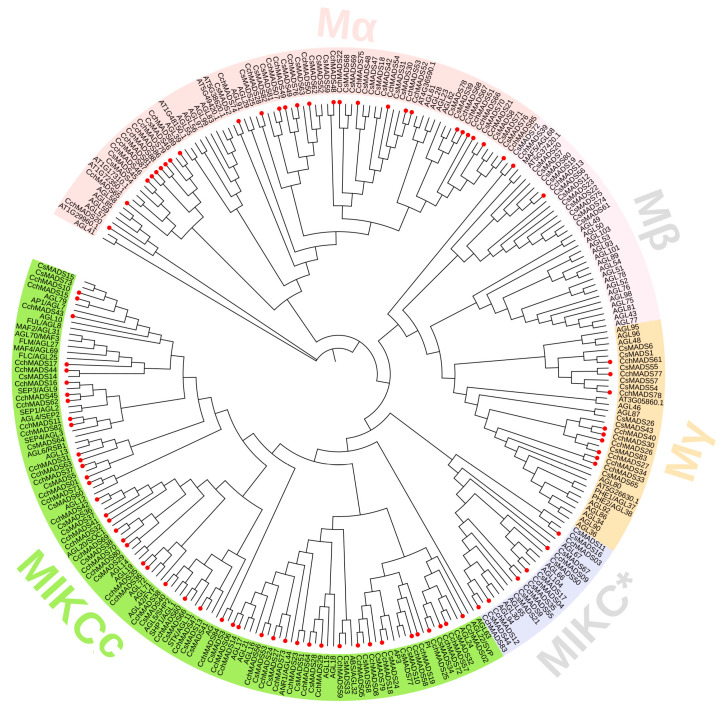
Phylogenetic tree comprising MADS-box proteins of *C. chekiangoleosa*, *C. sinensis*, and *A. thaliana*. The red circles represent CchMADS proteins. The light pink color indicates the Mβ subfamily, the light orange color indicates the Mγ subfamily, the light grey color indicates the MIKC* subfamily, the pink color indicates the Mα subfamily, and the light green color indicates the MIKC^C^ subfamily.

A phylogenetic tree was constructed based on the *MADS-box* genes of *A. thaliana*, *Camellia sinensis*, and *C. chekiangoleosa* (Figure 1), and the results showed that the CchMADS proteins could be divided into two categories: type I and type II. The type I proteins could be further divided into three subfamilies, namely, Mα (27), Mβ (2), and Mγ (9), and the type II proteins could be divided into two subfamilies, namely, MIKC^C^ (45) and MIKC* (6). The type of MIKC^C^ subfamily in *CchMADS* genes was shown in Table S1. 

Although the total number of *CchMADS* genes in *C. chekiangoleosa* was similar to that in *C. sinensis* (83) (Table S2), *C. sinensis* had more type I genes, and there were fewer type II MIKC^C^ genes than type II *CchMADS* genes. Moreover, there were significant differences in the number of genes in the Mβ and MIKC^C^ subfamilies. In addition, although the total number of *MADS-box* genes (106) in *A. thaliana* was much higher than that in *C. chekiangoleosa* and the number of genes in each type I subfamily was higher, the number of type II MIKC^C^ genes was lower in *A. thaliana* than in *C. chekiangoleosa*.

### 2.3. Gene Structure and Motif Analysis

**Figure 2:**
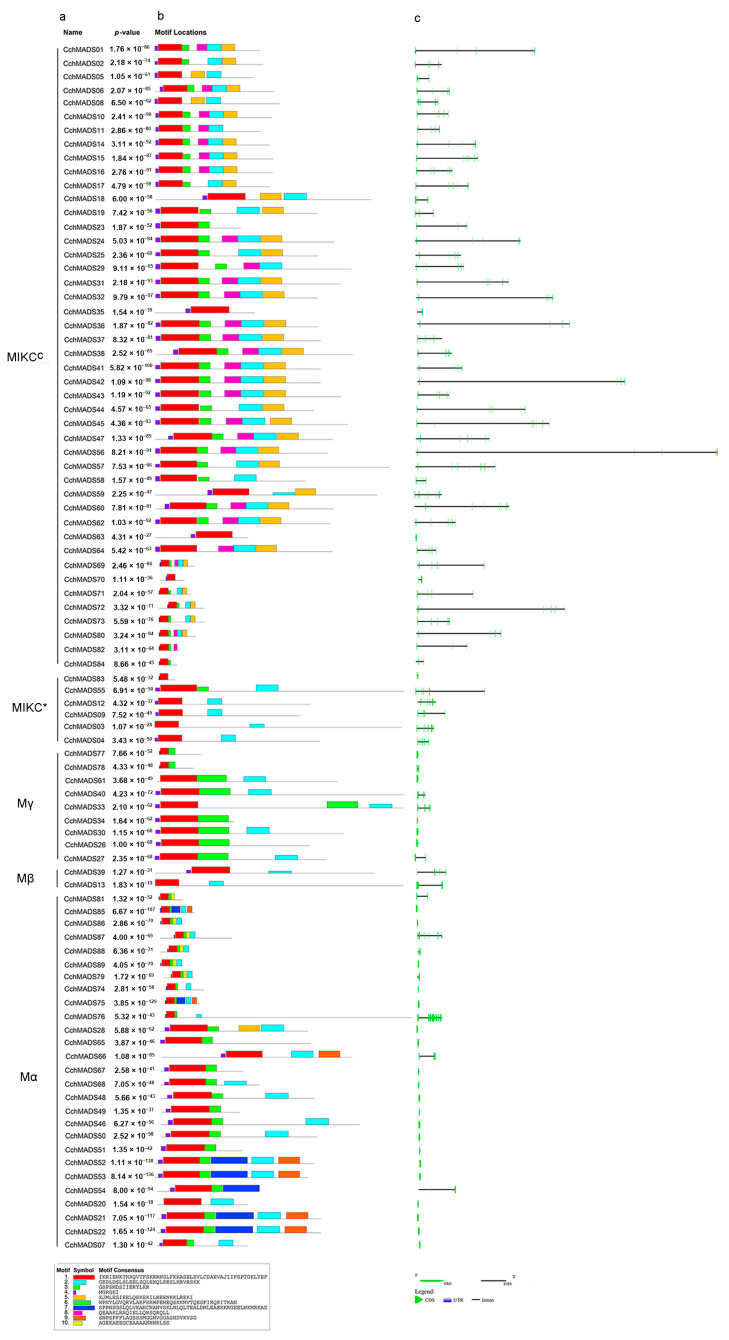
(**a**) The phylogenetic classification of the CchMADS proteins. (**b**) Motif analysis. The different colors of boxes represent different motif numbers. The length of a box indicates the motif length. (**c**) Structure of *CchMADS* genes.

Based on the results of our gene structure analysis (Figure 2c), it was found that among the 38 type I genes, 21 did not have introns. Four genes (*CchMADS33*, *CchMADS39*, *CchMADS76*, *CchMADS87*) contained 4-12 introns, and the remaining 13 genes contained 1~2 introns. Among the 51 type II genes, each contained at least one intron. Except for 8 genes (*CchMADS11*, *CchMADS23*, *CchMADS35*, *CchMADS63*, *CchMADS70*, *CchMADS82*, *CchMADS83*, *CchMADS84*) containing 1-4 introns, the number of introns of the remaining 43 genes were between 6 and 11. Overall, the average intron numbers of the type II genes (6.3) were much higher than those of the type I genes (1.6). In addition, we found that the length of introns for the different genes also varied greatly. The introns of *CchMADS54*, which were only 7 kb, were the longest among those of the type I genes. Among the type II genes, 39.2% of the introns were larger than 10 kb, and 11 genes, namely, *CchMADS24*, *CchMADS31*, *CchMADS32*, *CchMADS36*, *CchMADS42*, *CchMADS44*, *CchMADS45*, *CchMADS55*, *CchMADS56*, *CchMADS72*, and *CchMADS80*, had super large introns (≥15 kb). The length of these introns far exceeded that of the other genes.

In this study, a total of 10 conserved motifs of CchMADS proteins—motifs 1–10—were identified (Figure 2b). The results showed that motif 1, motif 2, and motif 4 were widely present in all CchMADS proteins. They were MADS domains, and motif 1 was the classic MADS domain. In addition, motif 3 only appeared in Mα and MIKC^C^. However, the type I proteins were quite different. Motif 6 was endemic to the Mγ subfamily, and motif 7, motif 9, and motif 10 were endemic to the Mα subfamily. The MIKC^C^ proteins were more conserved. Motifs 5 and 8 were specific to MIKC^C^ proteins. Motif 5 was a highly conserved K domain motif.

### 2.4. Chromosomal Localization and Duplication of CchMADS Genes

**Figure 3:**
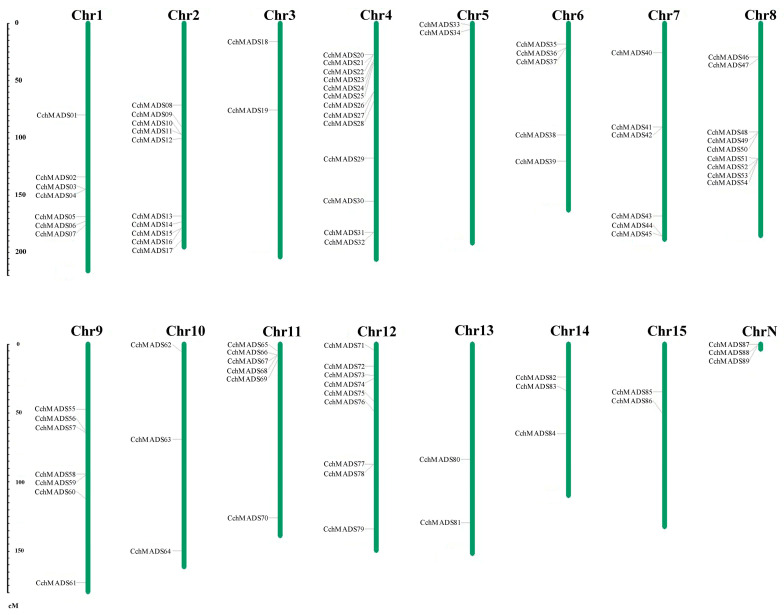
Chromosomal locations of *CchMADS* genes. The number of each chromosome is given above the lines.

Chromosomal localization was based on data within gff3 annotation files. We found that the 86 *CchMADS* genes were unevenly distributed on 15 chromosomes (Figure 3). These genes were named *CchMADS01* to *CchMADS86* according to their chromosomal localization. Only three *CchMADS* genes (*CchMADS87*, *CchMADS88*, *CchMADS89*) could not be mapped to any chromosome. The results showed that the proportion of genes on the 15 chromosomes was between 2.25% and 14.61%. Chromosomes 3, 5, 13, and 15 had the fewest *CchMADS* genes (2), whereas chromosome 4 had the most genes (13).

**Figure 4:**
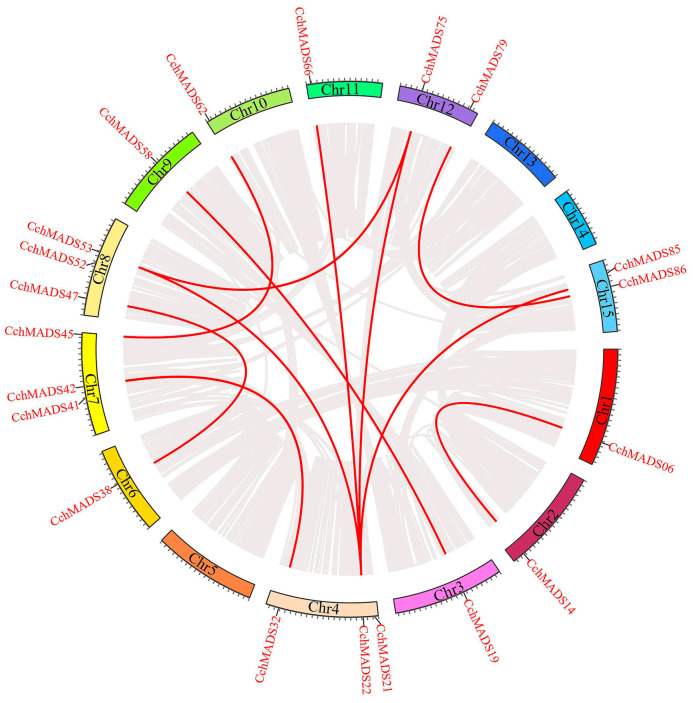
Fragment duplication of *CchMADS* genes, with the red lines connecting fragmentally duplicated gene pairs.

There was one pair of tandemly duplicated genes (*CchMADS41* and *CchMADS42*) (*CchMADS52* and *CchMADS53*) (Table S3) on chromosomes 7 and 8, respectively. One pair (*CchMADS88* and *CchMADS89*) of tandemly duplicated genes could not be mapped to any chromosome. In addition, 22.5% of segmentally duplicated genes were located on different chromosomes (Figure 4). Many duplicate sequences were detected on different chromosomes, which may be one of the driving forces of gene evolution. Their nonsynonymous (Ka) and synonymous (Ks) substitution rates were analyzed (Table S2), and it was found that all Ka/Ks values were less than 1, indicating that they evolved under purifying selection.

### 2.5. Cis-Acting Elements of MADS-Box Gene Family-Associated Promoters

To further study the regulatory mechanism of the *MADS-box* gene family in terms of the development of *C. chekiangoleosa*, cis-acting elements were analyzed (Figure S3). It was found that approximately 50 cis-acting elements could be effectively expressed, and the analysis revealed 21 elements with clear functions. Each gene had more than three light-responsive elements, which constituted the most abundant type (994), followed by hormone-responsive elements (509), including abscisic acid response elements, gibberellin response elements, methyl jasmonate (MeJA) response elements, etc. Most of the other response elements (303) were related to fruit and seed development, including circadian rhythm control, endosperm expression, and seed-specific regulation. This might mean that *CchMADS* genes play an important role in the reproductive growth of *C. chekiangoleosa*. The lowest number of cis-acting elements were involved in responses to abiotic stress (140), including drought stress, calli, etc.

### 2.6. Protein–Protein Interaction Network of CchMADSs

**Figure 5:**
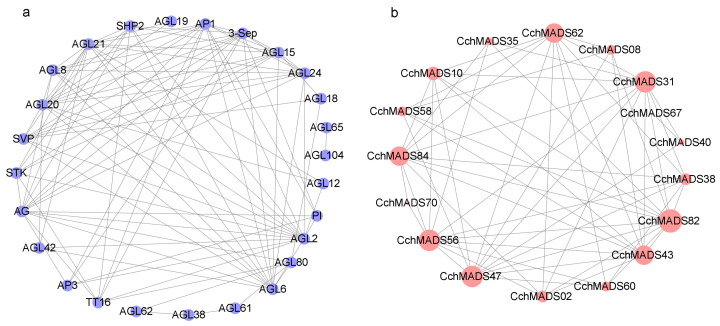
Protein–protein interactions between CchMADSs. (**a**) Blue color indicates *A. thaliana* genes homologous to the *CchMADS* genes. (**b**) Red color represents CchMADSs.

To elucidate the biological function and regulatory network of CchMADS proteins, a CchMADS protein–protein interaction network was predicted via the homologous MADS-box proteins of *A. thaliana* (Figure 5). Seventeen CchMADS proteins (CchMADS08, 31, 67, 40, 38, 82, 43, 60, 02, 47, 56, 70, 84, 58, 10, 35, 62, respectively) homologous to those in *A. thaliana* and 26 corresponding interacting functional genes (AP1, 3-Sep, AGL15, AGL24, AGL18, AGL65, AGL104, AGL12, PI, AGL2, AGL80, AGL6, AGL61, AGL38, AGL62, TT16, AP3, AGL42, AG, STK, SVP, AGL20, AGL8, AGL21, SHP2, AGL19, respectively) were identified. Among them, *CchMADS40* and *CchMADS67* were type I genes, and the rest belonged to the type II MIKC^C^ subfamily. In addition, with the exception of CchMADS67, the remaining CchMADS proteins were found to interact with more than one protein, and seven homologous proteins (CchMADS62, CchMADS31, CchMADS82, CchMADS43, CchMADS47, CchMADS56, CchMADS84) could interact with more than 10 other MADS proteins. Most of these proteins, such as AGL6, SVP, and TT16, are mainly related to the development of flowers. These proteins (such as SHP2, STK, and AGL38) are not only related to flower development but also affect fruit development, maturity, and seed dispersal.

### 2.7. Expression of CchMADS Genes in Different Tissues

**Figure 6:**
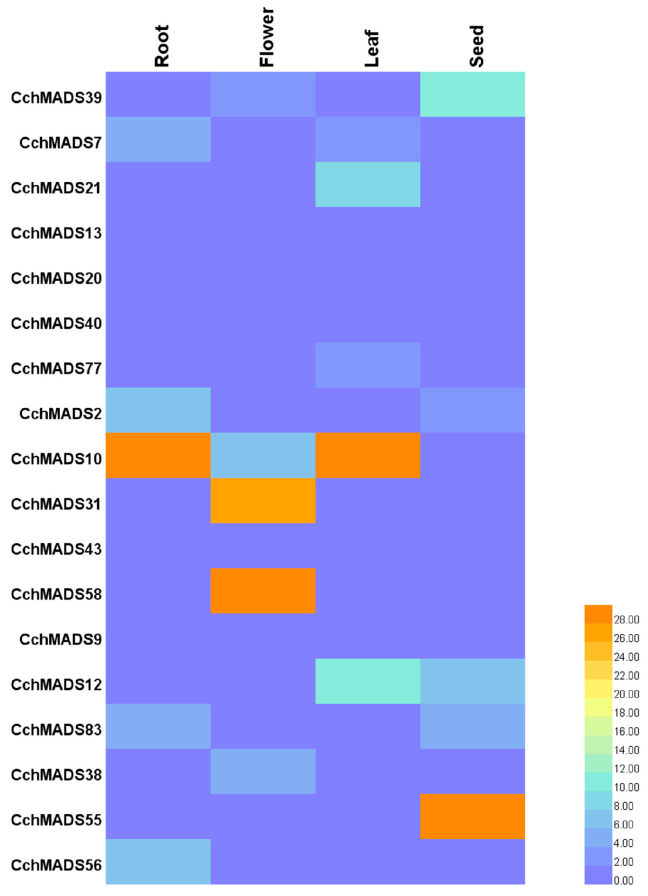
Relative expression of *CchMADS* genes in different tissues. The color scale represents relative expression levels from high (orange color) to low (blue color).

To further confirm the expression patterns of *CchMADS* genes in different organs and predict their potential role in plant growth and development, 18 genes were selected to explore their expression patterns in the roots, flowers, leaves, and seeds of *C. chekiangoleosa*. The information of primer sequence is shown in Table S4, the relative expression of *CchMADS* genes in four tissues is shown in Table S5. In Figure 6, the red block indicates high expression, and the blue indicates low expression. Overall, the expression of type I genes in all four tissues was lower than that of type II genes. Among the type I genes, the members of the Mβ and Mγ subfamilies (*CchMADS13*, *CchMADS20*, *CchMADS40*, *CchMADS77*) were expressed at very low levels in the four tissues. The genes of the Mα subfamily (*CchMADS39*, *CchMADS7*, *CchMADS21*) were slightly more highly expressed, especially *CchMADS39* in the seeds, whose expression was relatively high. Among the type II genes, the expression of *CchMADS12* in the leaves was relatively high, and *CchMADS10* was highly expressed in the roots and leaves. Notably, *CchMADS31* and *CchMADS58* showed high expression specifically in the flowers, and similarly, *CchMADS55* showed high expression specifically in the seeds, which might mean that these type II genes play important roles in the development of reproductive organs.

## 3. Discussion

### 3.1. Number and Characteristics of CchMADS Genes

After verification via the SMART program, 89 MADS-box gene family members (*CchMADS1* to *CchMADS89*) with intact MADS domains were ultimately identified in *C. chekiangoleosa*. However, these genes were not evenly distributed on the chromosomes. Zhang [29] found a similar number of genes in *C. sinensis* (83), which was more than that in *Salix suchowensis* (60) [30] and *Sesamum indicum* (57) [31] but less than that in *A. thaliana* (106) [32] and poplar (105) [33]. The genome sizes of these species varied (*C. sinensis*, 3 Gb; *C. chekiangoleosa*, 2.73 Gb; *S. suchowensis*, 356 Mb; *S. indicum*, 337 Mb; *A. thaliana*, 207 Mb; poplar, 431 Mb). In general, the number of gene family members is related specifically to genome size. However, some species seem to be unrelated, which may be the result of complex historical events, such as genome duplication, but the specific reasons need to be further explored.

Our constructed phylogenetic tree that referred to the classification of *A. thaliana* [32] revealed that the proportion of type II genes in *C. chekiangoleosa* (57.3%) was significantly higher than that in *C. sinensis* (43.4%) and *A. thaliana* (47.4%). This meant that type II *CchMADS* genes experienced a higher duplication rate or a lower gene loss rate after genome duplication. Although the proportion of type II genes was quite different, the MIKC^C^ subfamily (45 members) of *C. chekiangoleosa* had the most genes with homologs in *C. sinensis* and *A. thaliana*. This indicated that the genes of the MIKC^C^ subfamily were conserved between different species. In addition, through the subfamily classification in the MIKC^C^ group (Table S1), we found that the gene numbers of AGL17, SOC1, and SEP subfamilies in *C. chekiangoleosa* (8, 8, 5, respectively) was much higher than that of *C. sinensis* (3, 5, 2, respectively) [29]. There were no genes of the AGL6, AGL12, and Bsister subfamilies in *C. sinensis*. In previous research of *A. thaliana*, *AGL17*, *SOC1*, *SEP*, and *AGL6* affect the flower organs [19,34,35], *AGL12* affects root cell differentiation, and *Bsister* affects ovule and seed development [36,37]. These homologous genes in *C. chekiangoleosa* may also play a similar role, resulting in the characteristics of *C. chekiangoleosa* with large flowers and fruits. 

Other subfamilies were more varied, and the most obvious was the Mβ subfamily. The Mβ gene family of *C. chekiangoleosa* was 1/6 that of *C. sinensis* and poplar and only 1/10 that of *A. thaliana*. Gene loss might have occurred during the evolutionary process because the Mβ genes in *C. chekiangoleosa* failed to play an important role. Similar results were found in sesame and soybean [31,38]. Therefore, compared with other *CchMADS* genes, the MIKC^C^ genes may have undergone more duplication and differentiation in *C. chekiangoleosa*, while other *CchMADS* genes have been severely lost.

In addition, we found that the longest amino acid sequence of a MADS-box protein in *C. chekiangoleosa* was 1528 aa (CchMADS76), which was smaller than that in other species, such as *Malus domestica* (the longest being 593 aa) [22], *Setaria italica* L. (the longest being 477 aa) [28], and *Solanum lycopersicum* (the longest being 389 aa) [39]. In *C. sinensis* [29], a sequence of up to 2691 aa was found, which has not been reported in other species. Both genes belong to the Mα subfamily, which may be unique to the *Camellia* genus.

### 3.2. Expansion of the CchMADS Gene Family

Gene duplication was thought to be the product of errors in DNA replication and reconstruction. The copied genes may exert new functions and enhance the ability of plants to adapt to the environment [40,41]. In this study, both segmentally duplicated (18 pairs) and tandemly duplicated (3 pairs) gene pairs belonged to the Mα and MIKC^C^ subfamilies. The proportion of Mα subfamilies (54%) was relatively high. This is similar to findings of a study of soybean, where tandem duplication and segment duplication of type I genes were found to have occurred more frequently than those of type II genes [38]. This may be because type I genes originated and differentiated later than did type II genes, and type II genes were more conserved. Ka and Ks values are considered important indicators for studying the selection pressure or strength of protein-coding genes [42]. By calculating the Ka and Ks values, we found that the *CchMADS* genes evolved under the pressure of purifying selection. Segmental duplication and tandem duplication may be driving forces of gene family expansion and play an important role in functional gene diversity [43].

### 3.3. Intron Specificity of CchMADS Genes

The greater the number and length of introns, the more diverse the number of ways in which genes are spliced, thus affecting gene expression and protein activity [44,45,46]. The structure of type I genes of *C. chekiangoleosa* was simple, and most of them had no or only one intron, which is similar to the genes of *A. thaliana*. Compared with the type II genes of *C. sinensis*, the type I genes had more introns (average of 3.8). The reasons might be that this species has more type I genes and greater variation. The difference was that the number of introns (6.3) and the proportion of introns greater than 10 kb (39.2%) in type II *CchMADS* genes far exceeded those in *C. sinensis* (3.6 and 4.8%, respectively). A high proportion of type II genes was not found in other species, such as *A. thaliana* and *S. suchowensis*. Therefore, these type II genes may play important role in *C. chekiangoleosa*.

It has been suggested that genes with super large introns and transposons are more highly expressed [47,48]. Compared to only 3 MIKC^C^ genes in *C. sinensis*, more than 10 MIKC^C^ genes with super large introns (≥15 kb) in *C. chekiangoleosa* were found, but this was not the case in other species. MIKC^C^ genes play an important role in plant flower organ development, flowering time duration, and sex determination of male and female flowers [49]. The presence of super large introns in *Camellia* plant species, especially in *C. chekiangoleosa*, may have contributed to the specificity of *Camellia* plants. The morphology and size of the flowers and fruits of *C. chekiangoleosa* are quite different from those of other species, and the number of introns and longer length of its type II genes mean that the expression of genes is relatively diverse. These characteristics may affect the expression of MIKC^C^ genes in the reproductive organs of *C. chekiangoleosa* and play an important role in the formation of flower morphology and size.

### 3.4. Gene Expression and Potential Function

According to the results of qPCR, the *AGL6-like* gene *CchMADS31* and *PI-like* gene *CchMADS58* were highly expressed, specifically in flowers. The *AGL104-like* gene *CchMADS55* was expressed specifically in the seeds, and all of these genes were type II. It is believed that *AGL6* is involved in the gene regulation of floral meristems. *PI* affects the development of petals and stamens and is responsible for regulating the expression of genes associated with flower development [50,51]. *AGL104* double mutants have defects in pollen viability and pollen tube growth, resulting in delayed germination and reduced fertility [52]. Therefore, *CchMADS31* and *CchMADS58* may regulate the flower meristem of *C. chekiangoleosa* and affect the size of the petals, and *CchMADS55* may affect seed development. In addition, the type II genes *CchMADS10* and *CchMADS12* were also highly expressed in the roots and leaves, which meant that some type II genes not only play important roles in the reproductive organs of *C. chekiangoleosa* but also participate in the development of roots, leaves, and other tissues.

## 4. Materials and Methods

### 4.1. Identification of C. chekiangoleosa MADS-Box Genes

The *C. chekiangoleosa* genomic data were published by our team in 2022 and are publicly accessible at https://ngdc.cncb.ac.cn/gwh (accessed on 17 January 2022). The *A. thaliana* MADS-box protein sequences were obtained from The Arabidopsis Information Resource (TAIR) database (http://www.arabidopsis.org/, accessed on 10 January 2022) and utilized for BLASTP (E value = 1 × 10^−20^) searches against the sequences of proteins of *C. chekiangoleosa*. Moreover, the HMM profile of the MADS-box domain (Pfam accession PF00319) was downloaded from the Pfam database (http://pfam.xfam.org/, accessed on 11 January 2022) and then used to retrieve the MADS-box protein sequences from all the annotated genes of the *C. chekiangoleosa* genome via the HMMER program version 3.0 [53].

All the candidate protein sequences were assessed based on the presence of the conserved domain via the SMART program (http://smart.embl-heidelberg.de/, accessed on 20 January 2022) [54], Conserved Domain Database (CDD) search (https://www.ncbi.nlm.nih.gov/Structure/cdd/wrpsb.cgi, accessed on 21 January 2022), and PlantTFDB (http://planttfdb.gao-lab.org/, accessed on 22 January 2022) [55]. Sequences that incorrectly occupied or did not carry an entire domain were removed from the MADS-box genes. In addition, ExPASy (https://web.expasy.org/protparam/, accessed on 10 February 2022) [56] was used to analyze the physical and chemical properties of the MADS-box proteins, and Plant-mPLoc (http://www.csbio.sjtu.edu.cn/bioinf/plant-multi/, accessed on 12 February 2022) [57] was used for subcellular localization predictions.

### 4.2. Multiple Alignment and Phylogenetic Analysis

A sequence logo of the identified *C. chekiangoleosa* MADS-box genes was generated using WebLogo3 (http://weblogo.threeplusone.com, accessed on 16 February 2022) with the default parameters [58]. We subsequently used ClustalX version 2.1 to perform a multisequence alignment of the MADS-box domains, and ESPript 3.0 (https://espript.ibcp.fr/ESPript/cgi-bin/ESPript.cgi, accessed on 18 February 2022) was then used to visualize the resulting alignment.

A phylogenetic tree was constructed by the maximum likelihood (ML) method in MEGA version 11.0 [59]. The MADS-box protein sequences of *C. sinensis* were acquired from the article of Zhang [29]. Finally, the network profile of the phylogenetic tree was visualized by iTOLs version 6 (https://itol.embl.de/, accessed on 12 March 2022) [60].

### 4.3. Chromosomal Localization and Gene Structure Analysis

The distribution of the 89 MADS-box genes and gene density were visualized using TBtools version 1.098745 [61]. The conserved motifs were analyzed using MEME (http://meme-suite.org/, accessed on 20 March 2022) [62]. The parameters were set to a repeat motif site of any number, a maximum number of motifs of 10, and a width of each motif ranging from 6 to 60 residues. An exon/intron map was constructed in the Gene Structure Display Server program (http://gsds.cbi.pku.edu.cn/, accessed on 21 March 2022) [63].

### 4.4. Gene Duplication and Promoter Cis-Acting Regulatory Element Analysis

MCScanX [64] was used to analyze gene duplication. Multi-sequence and BLASTp alignments (E-value = 1 × 10^−20^) were performed to obtain the similarities between these *CchMADS* genes. The major criteria used for analyzing potential gene duplications included the following: (a) the length of sequence that can be aligned covers 75% of the longer gene, and (b) the similarity of aligned regions covers 75% [65]. When a duplicated gene pair constituted two consecutive genes on the same chromosome, it was considered a tandemly duplicated gene pair. The Ka and Ks values were determined via KaKs_Calculator [42].

The upstream regions (2000 bp) of the start codon (ATG) of the MADS-box genes were used as the gene promoter sequence and retrieved from the *C. chekiangoleosa* genome, and its cis-acting elements were analyzed using PlantCARE (http://bioinformatics.psb.ugent.be/webtools/plantcare/html/, accessed on 2 April 2022) online software. The results were visualized by TBtools.

### 4.5. Protein–Protein Interaction Network Analysis

The MADS-box protein interaction network of *C. chekiangoleosa* was analyzed using the online website String (https://string-db.org/, accessed on 12 April 2022). The protein interaction network was visualized using Cytoscape version 3.9.1 software [66].

### 4.6. Plant Materials and Expression Analysis in Different Tissues

The experimental qPCR materials, which included 5-year-old live seedlings, were derived from the germplasm resource garden of the Zhongshan Botanical Garden of Jiangsu Province (118°69′ N, 32°51′ E). Seeds were collected in August 2021, and roots, flowers, and leaves were collected in March 2022. Total RNA was isolated using an RNA kit (RNAprep Pure Plant Kit, Tiangen, Beijing, China), the extraction procedures could be found in the manufacturer’s instructions. The quality and concentration of RNA samples were determined by the NanoDrop 2000 c spectrophotometer (Thermo Scientific, Wilmington, DE, USA) and 1% agarose gel electrophoresis. cDNA was synthesized from 1000 ng of total RNA in a 20 μL reaction volume using PrimeScriptTM RT Master Mix (TaKaRa, Dalian, China). We took 3 different biological replicates per tissue and froze them in liquid nitrogen. Then, we stored them in a −80 °C freezer. The 2^−ΔΔCT^ method was used to calculate the relative expression in the various tissues. The expression levels were log10 standardized, and Heml version 1.0 software (http://hemi.biocuckoo.org/down.php, accessed on 20 May 2022) was used to construct an expression profile heatmap of the *CchMADS* genes.

## 5. Conclusions

In this study, a total of 89 *MADS-box* genes were identified in *C. chekiangoleosa*. Fragment duplication and tandem duplication were the driving forces for the expansion of *MADS-box* family members. These genes could be divided into type I and type II, of which there were 38 and 51 genes, respectively. The proportion of type II genes in *C. chekiangoleosa* was higher than that in the other species analyzed. Through phylogenetic, conserved motif, and other analyses, we found that the structure of the type II genes is more conserved than that of the type I genes. The presence of superlong amino acid sequences may be an important feature of the *Camellia* genus. Compared with type I genes, type II genes are present in a higher proportion, have a higher number of introns, and have longer introns in the genome of *C. chekiangoleosa*. The superlong introns of many genes of the MIKC^C^ subfamily may indicate increased gene expression. Further qPCR analysis found that the overall expression level of the type II genes was significantly higher than that of the type I genes. Some type II genes were highly expressed specifically in the reproductive organs, indicating that these genes may be involved in regulating their developmental process. In addition, we found evidence of the expression of the *MADS-box* genes in the roots and leaves. This study provides additional information for the functional characterization of the *MADS-box* gene family. At the same time, it establishes an important foundation for the in-depth study of reproductive organ development and other related genes of *C. chekiangoleosa*.

## Figures and Tables

**Table 1:** Amino acid composition and physiochemical properties of CchMADS proteins.

Gene Name	Sequence ID	Location	Length (aa)	M_W_ (kDa)	pI	Subcellular Localization	Type	Intron
CchMADS01	Cole01G001968.1	Chr1	222	25.823	6.47	Nucleus	MIKC^C^	6
CchMADS02	Cole01G003080.1	Chr1	229	25.997	5.75	Nucleus	MIKC^C^	7
CchMADS03	Cole01G003276.1	Chr1	522	58.723	5.46	Nucleus	MIKC*	11
CchMADS04	Cole01G003279.1	Chr1	349	39.503	4.96	Nucleus	MIKC*	10
CchMADS05	Cole01G003924.1	Chr1	208	24.266	9.48	Nucleus	MIKC^C^	6
CchMADS06	Cole01G004072.1	Chr1	252	29.436	8.76	Nucleus	MIKC^C^	6
CchMADS07	Cole01G004142.1	Chr1	193	21.333	9.37	Nucleus	Mα	0
CchMADS08	Cole02G001598.1	Chr2	263	29.794	9.01	Nucleus	MIKC^C^	5
CchMADS09	Cole02G002131.1	Chr2	307	34.614	5.54	Nucleus	MIKC*	8
CchMADS10	Cole02G002392.1	Chr2	246	28.233	8.93	Nucleus	MIKC^C^	7
CchMADS11	Cole02G002395.1	Chr2	223	25.637	9	Nucleus	MIKC^C^	4
CchMADS12	Cole02G002503.1	Chr2	329	37.934	6.64	Nucleus	MIKC*	8
CchMADS13	Cole02G003948.1	Chr2	524	59.964	6.19	Nucleus	Mβ	1
CchMADS14	Cole02G004095.1	Chr2	242	28.203	8.44	Nucleus	MIKC^C^	6
CchMADS15	Cole02G004285.1	Chr2	249	28.652	9.02	Nucleus	MIKC^C^	7
CchMADS16	Cole02G004286.1	Chr2	248	29.003	7.08	Nucleus	MIKC^C^	7
CchMADS17	Cole02G004715.1	Chr2	240	27.737	7.75	Nucleus	MIKC^C^	6
CchMADS18	Cole03G000406.1	Chr3	289	33.388	8.89	Nucleus	MIKC^C^	6
CchMADS19	Cole03G001914.1	Chr3	216	25.379	6.77	Nucleus	MIKC^C^	6
CchMADS20	Cole04G000929.1	Chr4	121	13.591	9.83	Nucleus	Mα	0
CchMADS21	Cole04G000931.1	Chr4	219	24.905	9.49	Nucleus	Mα	0
CchMADS22	Cole04G000947.1	Chr4	219	24.247	9.08	Nucleus	Mα	0
CchMADS23	Cole04G001061.1	Chr4	114	12.833	9.61	Nucleus	MIKC^C^	2
CchMADS24	Cole04G001062.1	Chr4	239	27.475	6.41	Nucleus	MIKC^C^	7
CchMADS25	Cole04G001125.1	Chr4	218	25.004	8.61	Nucleus	MIKC^C^	7
CchMADS26	Cole04G001870.1	Chr4	206	24.217	9.2	Nucleus	Mγ	1
CchMADS27	Cole04G001878.1	Chr4	230	26.646	9.55	Nucleus	Mγ	1
CchMADS28	Cole04G002096.1	Chr4	197	22.197	7.69	Nucleus	Mα	0
CchMADS29	Cole04G003100.1	Chr4	262	30.047	6.13	Nucleus	MIKC^C^	7
CchMADS30	Cole04G003823.1	Chr4	252	28.918	9.77	Nucleus	Mγ	0
CchMADS31	Cole04G004469.1	Chr4	248	28.181	8.83	Nucleus	MIKC^C^	7
CchMADS32	Cole04G004470.1	Chr4	217	24.824	6.47	Nucleus	MIKC^C^	6
CchMADS33	Cole05G000003.1	Chr5	332	38.973	10.14	Nucleus	Mγ	4
CchMADS34	Cole05G000013.1	Chr5	106	12.359	10.12	Nucleus	Mγ	0
CchMADS35	Cole06G000488.1	Chr6	131	14.624	9.16	Nucleus	MIKC^C^	2
CchMADS36	Cole06G000572.1	Chr6	213	24.628	8.8	Nucleus	MIKC^C^	6
CchMADS37	Cole06G000576.1	Chr6	217	24.795	7.05	Nucleus	MIKC^C^	7
CchMADS38	Cole06G002198.1	Chr6	259	29.666	9.2	Nucleus	MIKC^C^	8
CchMADS39	Cole06G002899.1	Chr6	289	32.705	8.65	Nucleus	Mβ	10
CchMADS40	Cole07G000640.1	Chr7	326	37.273	9.82	Nucleus	Mγ	2
CchMADS41	Cole07G001913.1	Chr7	217	25.053	6.39	Nucleus	MIKC^C^	6
CchMADS42	Cole07G001920.1	Chr7	217	25.042	6.18	Nucleus	MIKC^C^	6
CchMADS43	Cole07G004098.1	Chr7	244	28.547	8.44	Nucleus	MIKC^C^	7
CchMADS44	Cole07G004743.1	Chr7	208	23.709	9.49	Nucleus	MIKC^C^	6
CchMADS45	Cole07G004746.1	Chr7	253	28.615	8.74	Nucleus	MIKC^C^	7
CchMADS46	Cole08G000724.1	Chr8	261	28.432	6.76	Nucleus	Mα	0
CchMADS47	Cole08G000781.1	Chr8	233	26.711	9.78	Nucleus	MIKC^C^	8
CchMADS48	Cole08G002218.1	Chr8	202	22.749	8.45	Nucleus	Mα	0
CchMADS49	Cole08G002233.1	Chr8	103	11.521	9.56	Nucleus	Mα	0
CchMADS50	Cole08G002238.1	Chr8	205	23.551	6.72	Nucleus	Mα	0
CchMADS51	Cole08G002611.1	Chr8	107	11.853	9.96	Nucleus	Mα	1
CchMADS52	Cole08G002612.1	Chr8	219	24.053	9.32	Nucleus	Mα	0
CchMADS53	Cole08G002613.1	Chr8	211	23.132	9.12	Nucleus	Mα	0
CchMADS54	Cole08G002614.1	Chr8	141	15.725	9.94	Nucleus	Mα	1
CchMADS55	Cole09G000962.1	Chr9	342	38.905	5.65	Nucleus	MIKC*	10
CchMADS56	Cole09G001241.1	Chr9	237	27.304	9.38	Nucleus	MIKC^C^	6
CchMADS57	Cole09G001278.1	Chr9	323	35.838	6.85	Nucleus	MIKC^C^	9
CchMADS58	Cole09G001941.1	Chr9	208	24.372	9.11	Nucleus	MIKC^C^	6
CchMADS59	Cole09G001965.1	Chr9	306	35.993	9.35	Nucleus	MIKC^C^	9
CchMADS60	Cole09G002445.1	Chr9	245	28.275	9.22	Nucleus	MIKC^C^	9
CchMADS61	Cole09G004317.1	Chr9	250	28.592	4.92	Nucleus	Mγ	0
CchMADS62	Cole10G000146.1	Chr10	242	27.683	8.96	Nucleus	MIKC^C^	7
CchMADS63	Cole10G001610.1	Chr10	128	14.444	9.9	Nucleus	MIKC^C^	1
CchMADS64	Cole10G003622.1	Chr10	244	27.614	8.24	Nucleus	MIKC^C^	7
CchMADS65	Cole11G000155.1	Chr11	208	23.141	5.22	Nucleus	Mα	0
CchMADS66	Cole11G000294.1	Chr11	263	30.297	9.35	Nucleus	Mα	2
CchMADS67	Cole11G000296.1	Chr11	114	12.381	10.48	Nucleus	Mα	1
CchMADS68	Cole11G000299.1	Chr11	136	15.199	7.92	Nucleus	Mα	1
CchMADS69	Cole11G000477.1	Chr11	213	24.686	7.66	Nucleus	MIKC^C^	6
CchMADS70	Cole11G003071.1	Chr11	152	17.325	9.32	Nucleus	MIKC^C^	2
CchMADS71	Cole12G000125.1	Chr12	199	22.838	7.02	Nucleus	MIKC^C^	6
CchMADS72	Cole12G000456.1	Chr12	279	31.438	9	Nucleus	MIKC^C^	8
CchMADS73	Cole12G000684.1	Chr12	282	32.291	8.63	Nucleus	MIKC^C^	7
CchMADS74	Cole12G000760.2	Chr12	246	26.972	6.54	Nucleus	Mα	0
CchMADS75	Cole12G001299.1	Chr12	219	24.101	9.25	Nucleus	Mα	0
CchMADS76	Cole12G001525.1	Chr12	1528	176.716	9.12	Nucleus	Mα	12
CchMADS77	Cole12G002412.1	Chr12	257	29.344	5.7	Nucleus	Mγ	0
CchMADS78	Cole12G002414.1	Chr12	213	24.174	5.3	Nucleus	Mγ	1
CchMADS79	Cole12G003350.1	Chr12	184	20.516	6.45	Nucleus	Mα	1
CchMADS80	Cole13G001797.1	Chr13	226	25.852	6.09	Nucleus	MIKC^C^	6
CchMADS81	Cole13G003210.1	Chr13	149	17.171	10.44	Nucleus	Mα	1
CchMADS82	Cole14G000617.1	Chr14	124	14.314	9.67	Nucleus	MIKC^C^	3
CchMADS83	Cole14G000851.1	Chr14	102	12.018	9.78	Nucleus	MIKC*	1
CchMADS84	Cole14G001528.1	Chr14	113	13.234	9.84	Nucleus	MIKC^C^	2
CchMADS85	Cole15G000774.1	Chr15	219	24.682	9.72	Nucleus	Mα	0
CchMADS86	Cole15G000995.1	Chr15	141	15.648	6.32	Nucleus	Mα	0
CchMADS87	Cole00G064455.1	ChrN	439	48.72	5.9	Nucleus	Mα	7
CchMADS88	Cole00G064458.1	ChrN	184	20.505	5.75	Nucleus	Mα	1
CchMADS89	Cole00G064466.1	ChrN	141	15.536	6.32	Nucleus	Mα	0

M_W_: molecular weight (kDa), PI: isoelectric point.

## Data Availability

Not applicable.

## Supplementary Materials

The following supporting information can be downloaded at: https://www.mdpi.com/article/10.3390/ijms24043434/s1.
