# Supplementary material for: Gene Structural Specificity and Expression of MADS-Box Gene Family in Camellia chekiangoleosa
Source: Int J Mol Sci. 2023 Feb 8;24(4):3434. doi: 10.3390/ijms24043434 (PMC9960327; doi:10.3390/ijms24043434)
Supplement: Supplementary file 1 [file ijms-24-03434-s001.zip › ijms-2119342-supplementary.pdf]

Supplementary Materials

Table S1. The type of $MIKC^C$ subfamily in *CchMADS* genes.

Genes	Type
CchMADS10	AP1
CchMADS15	AP1
CchMADS43	AP1
CchMADS17	FLC
CchMADS44	FLC
CchMADS16	SEP
CchMADS45	SEP
CchMADS62	SEP
CchMADS11	SEP
CchMADS82	SEP
CchMADS31	AGL6
CchMADS63	AGL6
CchMADS37	AGL6
CchMADS01	AGL6
CchMADS71	AGL12
CchMADS42	SOC1
CchMADS41	SOC1
CchMADS32	SOC1
CchMADS69	SOC1
CchMADS70	SOC1
CchMADS80	SOC1
CchMADS35	SOC1
CchMADS36	SOC1
CchMADS38	AG/STK
CchMADS60	AG/STK
CchMADS47	AG/STK

CchMADS84	AG/STK
CchMADS06	AGL17
CchMADS14	AGL17
CchMADS56	AGL17
CchMADS24	AGL17
CchMADS73	AGL17
CchMADS23	AGL17
CchMADS64	AGL17
CchMADS29	AGL17
CchMADS59	Bsister
CchMADS05	AP3
CchMADS08	AP3
CchMADS18	AP3
CchMADS58	PI
CchMADS19	PI
CchMADS25	SVP
CchMADS72	SVP
CchMADS57	SVP
CchMADS02	SVP

Table S2. MADS-box transcription factor statistics for three species.

**Species**	**Type I**	**Mα**	**Mβ**	**Mγ**	**Type Ⅱ**	**MIKC^{C}**	**MIKC***	**Total**
Camellia chekiangoleosa	38	27	2	9	51	45	6	89
Camellia sinensis	47	26	12	9	36	29	7	83
Arabidopsis thaliana	61	25	20	16	45	39	6	106

Table S3. Ka, Ks and Ka/Ks of duplication pairs of MADS-box gene family in *C. chekiangoleosa*.

**Duplicated gene pairs**	**Non synonymous (*Ka*)**	**Synonymous (*Ks*)**	***Ka*/*Ks***	**Duplicated type**
CchMADS41& CchMADS42	0.01780	0.04429	0.40198	tandem

Duplicated gene pairs	Non synonymous (*Ka*)	Synonymous (*Ks*)	*Ka*/*Ks*	Duplicated type
CchMADS52& CchMADS53	0.02553	0.05620	0.45419	tandem
CchMADS06& CchMADS14	0.09326	0.41192	0.22639	segmental
CchMADS19& CchMADS58	0.11080	0.36441	0.30406	segmental
CchMADS21& CchMADS66	0.02774	0.06055	0.45809	segmental
CchMADS21& CchMADS85	0.05253	0.08801	0.59683	segmental
CchMADS22& CchMADS52	0.07317	0.23215	0.31516	segmental
CchMADS22& CchMADS53	0.09041	0.27007	0.33476	segmental
CchMADS22& CchMADS75	0.07221	0.22607	0.31941	segmental
CchMADS32& CchMADS41	0.11772	0.55412	0.21244	segmental
CchMADS32& CchMADS42	0.11640	0.55053	0.21144	segmental
CchMADS45& CchMADS62	0.03754	0.35991	0.10431	segmental
CchMADS47& CchMADS38	0.21223	1.68207	0.12617	segmental
CchMADS52& CchMADS75	0.04142	0.07435	0.55713	segmental
CchMADS53& CchMADS75	0.04802	0.11515	0.41699	segmental
CchMADS79& CchMADS86	0.02180	0.12272	0.17761	segmental
CchMADS79& CchMADS88	0.03926	0.09059	0.43339	segmental
CchMADS79& CchMADS89	0.03628	0.08043	0.45111	segmental
CchMADS86& CchMADS88	0.01550	0.11070	0.14006	segmental
CchMADS86& CchMADS89	0.03143	0.10948	0.28712	segmental
CchMADS88& CchMADS89	0.02985	0.04722	0.63210	tandem

Table S4. The information of primer sequence.

Gene ID	Primer Sequence (5′~3′)
CchMADS02	AGGAGCTCCCAGGGTTAAGT
	TAGTCTTGTGGAGGGCTGGT
CchMADS07	CCGGAGGTCGCAGAAAGATT

Gene ID	Primer Sequence (5′~3′)
	TGGGGCTTTCGTAAAGGGAC
CchMADS39	ACTAGCTTCGCAAGCAATGG
	CCAGCATTTCTGCTTCTTGC
CchMADS56	ACGCGGAGGTAGGAGTTATG
	GACCTCGGAACATGGGTTTG
CchMADS83	TGCAAGGAGATGCCTTGGAT
	TGGTGGAATGCTCAGATGGA
CchMADS21	TTGGCCACCCTAATGTGGAT
	GCACGCTAGCATTACGATGA
CchMADS13	TGGCCCCAAAATCCCAATGT
	AGGCCAATAAGCCCCTTTGA
CchMADS40	AACGCGATCTTGAGTGGTTG
	TGCATTCCATTGCCAAGCAT
CchMADS09	TGTTCTCTCCCTCTGGTCGT
	CCATGGTTCTGGTGCGTAGAT
CchMADS20	TAAGCTTCTTGAAACGCCGC
	AGCACAAGCATGACAGCAAC
CchMADS77	ACAGTCACGAGGAAGCTGAT
	GACCCTTTGTGTGAGGAAGC
CchMADS10	AAAACGCCGTTCTGGACTTC
	CTGCCTGGTAAGCACCACTTC
CchMADS12	AAGTTGGAGAGCCTTGAAGAATTA
	ATCTCCGTTTTGCCAGGACC
CchMADS55	TTTGGATTGGATGCCACAGC
	TGGTGGTCCTAGCATGTTGT
CchMADS31	CAACAGTGTGGAACGGGAAA
	CCAAGCAAGTGCCTTTGAGT
CchMADS58	TGACAGCTTGCAGATTGAGC
	TCTCCGACTGTTTGTAGCGA
CchMADS43	AGCAGAGCAGTCTCAATGGG
	CAAGCTCATTCCTCCTCCCT
CchMADS38	AGCCATTAGTCGGGTTCGAT
	GCTCTTGCTCTTGCTGTTGT
Actin	GTGGTTGTGAATGGAGGGCA
	AGGCCAGGAGAGCCATTACA

Table S5. Relative expression of *CchMADS* genes in four tissues.

Gene ID	Root	Flower	Leaf	Seed	Type
CchMADS39	1.86016	2.43381	1.34171	10.49596	Mα
CchMADS7	4.25414	0.81469	2.84748	1.00000	Mα
CchMADS21	0.80100	0.54500	9.07366	1.53065	Mα
CchMADS13	0.17205	0.04087	0.54438	0.00478	Mβ
CchMADS20	0.17505	0.38564	1.56448	0.03156	Mα
CchMADS40	0.31483	0.10047	1.59540	0.08727	Mγ
CchMADS77	1.40529	0.46597	2.62021	0.01080	Mγ
CchMADS2	6.61100	0.00046	0.00725	2.17670	$MIKC^{C}$
CchMADS10	44.38096	7.18665	64.92419	0.01003	$MIKC^{C}$
CchMADS31	0.03828	27.23367	0.04143	0.06458	$MIKC^{C}$
CchMADS43	0.00111	1.67518	0.09225	0.00469	$MIKC^{C}$
CchMADS58	0.15208	102.41759	0.05497	0.01798	$MIKC^{C}$
CchMADS9	0.40245	0.11796	1.47906	0.10689	MIKC*
CchMADS12	1.89194	1.33915	10.40124	5.97585	MIKC*
CchMADS83	5.18004	1.29069	1.79746	4.29226	MIKC*
CchMADS38	0.44984	5.28716	0.00000	0.09858	$MIKC^{C}$
CchMADS55	0.22841	0.01033	0.00000	210.87420	MIKC*

Gene ID	Root	Flower	Leaf	Seed	Type
CchMADS56	7.53114	0.00551	0.00000	0.00000	$MIKC^{C}$
Average	4.50936	9.73652	5.46584	13.15460	

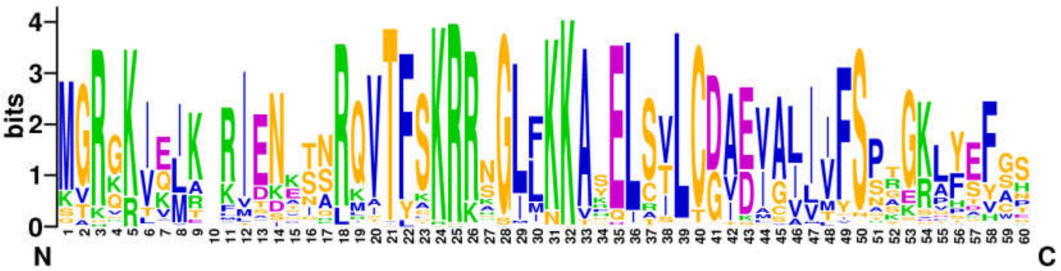

Figure S1. Conserved domain sequence icon of MADS-box protein.

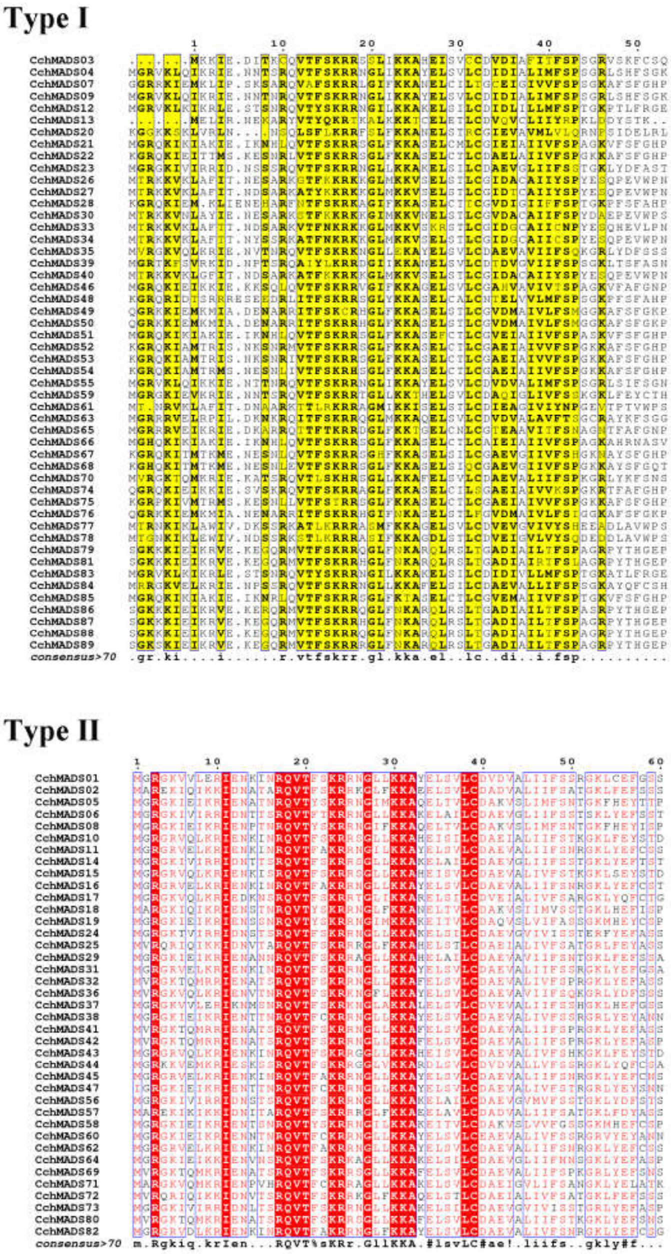

Figure S2. Multi-alignment of MADS-box domain.

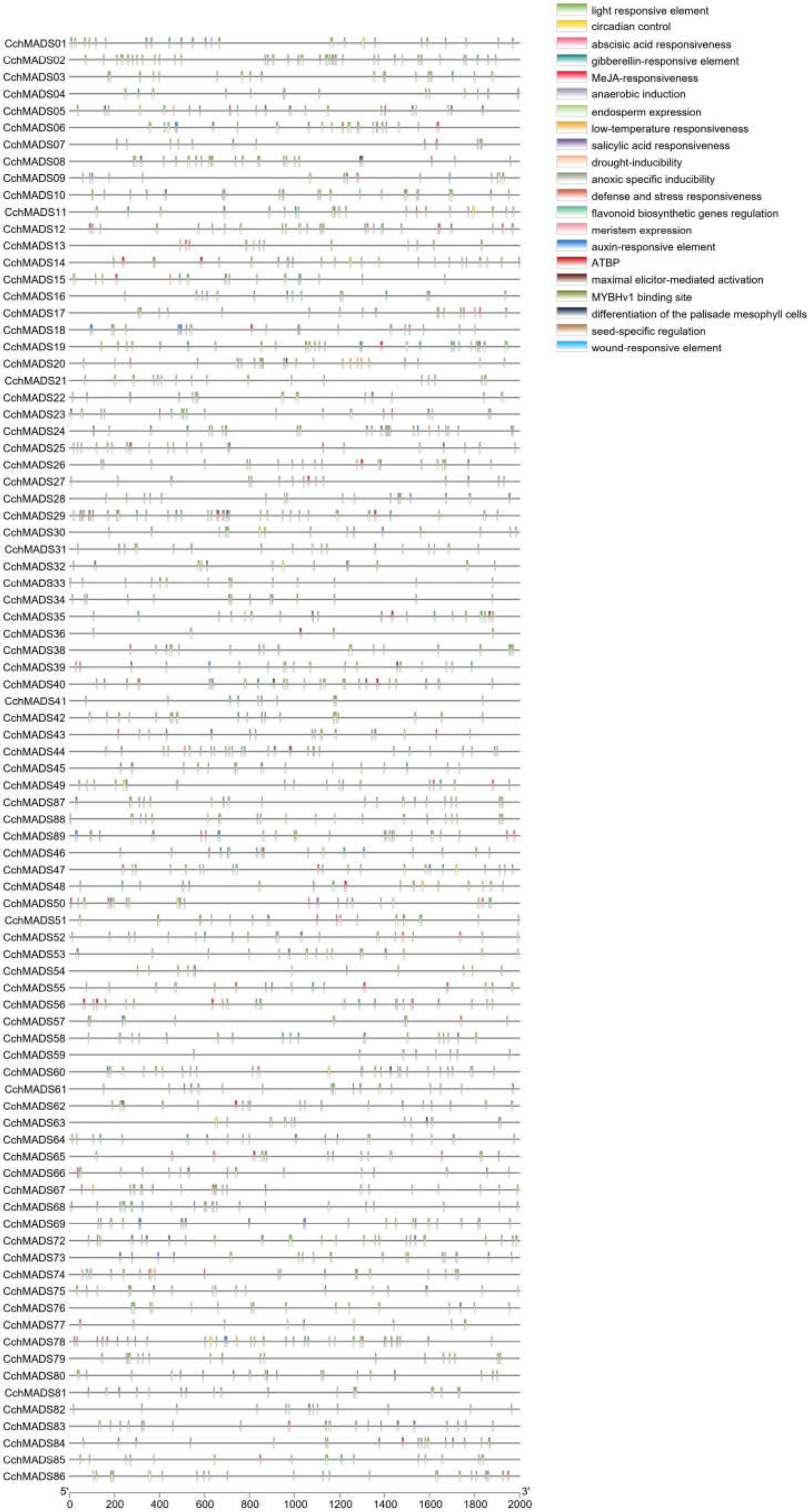

Figure S3. Promoter cis-acting element in *C. chekiangoleosa*. Different colors represent different types of cis-acting elements.

File S1. MADS-box protein sequence of *C. chekiangoleosa*.

>CchMADS01

MGRGKVVLERIENKINRQVTFSKRRNGLLKKAYELSVLCDVDVALIIFSSRGKLCEFGSSNISQIIER
YRQYCYTTQDDNPLEHEPQLFRELTKLKADYESLQHSQRHLLGEDLAPLDMKELLNLEEQLDGT
LSKARQKKTQMMLERMEALREKERDLEERNKQLKTKLEEVEEHIREIQSLRSVNATVGSNGIQVQ
ASQSNPVEIETSLQIGYIYFFCPSK

>CchMADS02

MAREKIQIKKIDNATARQVTFSKRRKGLFKKAEELSVLCDADVALIIFSATGKLFEFSSSSMTEILER
RNLHSKNLDKLDQPSLELQLVENSNFSRLSKEVAEKSHQLRQMRGEELPGLSIEELQQLERSLEAG
LSHVIEKKGEKIMKEISNLQQKEMELMEENERLRQQVKEVSNARKHVAVVTDSENMYINEEGQSS
ESVNICNSTSPPQDYDSSVPSLKLGLPYSG

>CchMADS03

MKKIEDITKCQVTFSKRRSSLIKKAHEISVCCDVDIAFITFSPSGRVSKFCSQRRIEDLIHRYINLPVDK
RFTYVTIPTLHFVNFHIKNVQEKQEKIHQLDRIKGDSSKLQYLEKQLDSLSLSLTILIYLDYELLPEQE
PSLHQLMWCERNLKQSLEKVVARKNALSGSYSSLQSQPNTQVEMENQGGMPLSCYIDGRDLSW
VPKSPPSRQLLMQLDPWISPYSAAVRESIFQGITDQIKGTPVSSQTPGSLTSHISIKAFSTKNQSGNSS
GAQETLITLPTQQPSVDIEFSSILSNHGGVSDSSSKQNHLNKQDIIISLAQSKSNHKNGNWFASGNP
NCSKSLSVTQPPNVARNISLPINGSFCTASASSATTTTTAKGLNLDELGLYQMEIGMEKYNPENDQ
TITTSKWFNPKTTISQHNNVKQIDDQCEIDGNNNNSASNNDTKDSNTESGFVVMDQSIDMDPLH
KFPEVPSFDDFSLENILEDNNLWTKRIQESGLWEWDDLVMADNMNFEDLEKFFD

>CchMADS04

MGRVKLQIKRIENNTSRQVTFSKRRNGLIKKAYELSVLCDIDIALIMFSPSGRLSHFSGKRRIEDVLS
RYINLADHDRGGILQNREYLITTLKKLKNEDDMALEFASPTELNSNAEELQQEINNLQHQLQMA
EEQLSIFEPDILGFTSMEELESCEKNLLESMNHVIQRKKYLMGDHLSSFDHQSMQMNSLGQMFFE
AQVGMPTTSFQNDLVSWMPQNGDNQNHIFGSSHDHPCIPLRNDSPTTIYNSLSHATDENGEPNS
ISGCPIHSPNDDHSLSQWHHACTSSLFSSLMPSTSFPQNDMEVSPPDIVAAEQHHQQVEATSTCPE
VPSNEEEGNFENKLPELNVESS

>CchMADS05

MGRGKIEIKRIENATNRQVTYSKRRNGIMKKAQELTVLCDAKVSLIMFSNTGKFHEYTTPNIATKK
IYDQYQKNLGIDLWSTHYERMQGHLKKLKEINNKLRREIGQRVGGEDLNDLSIQELCGLEQNMA
ASLTDVRQRKYHVLKTQTETYKKKVRSMEERHGNLLLNFEAKCEDPQFGTLFENDRDYDYAFRL
HRGGGFGSNELRLA

>CchMADS06

MIQTLESRTVRMGRGKIVIRRIDNTTSRQVTFTKRRNGLLKKAKELAILCDAEVGLIIFSSTSKLYEFS
STSMKSVIERYNKSNEERQLHNQVSEVKFWQREAEILRQQLHNLQENYRQLMGEELYGLSVHDL
QKLENKLEMSLRGVRMQKDQVLMDEIQELSQKENFMHQENVELYKKANLIRQENMELCKKGQ
NFLPQIYGTRDVTAANGSTQNPHGFSIGEDSYVPINLQLSQPEQQSHEMPARETRSQ

>CchMADS07

MARKMAGGRRKIEMKLIPSKSARQVAFSKRRLGIFKKANELCILTGCEIGIVVFSPSGKAFSFGHPS
VDTIVQRSLYESPMPAAIDQDSHGSIVPALCQEYTEMCRQLGAEKRRGKELKESAIKCQRPYWLD
APIHELNLDQVLDSKKCMEELRAKIAKRVNELSVEGSASNLASSAKFVRGIDLNVAIPDAI

>CchMADS08

MGRGKIEIKRIENPTNRQVTYSKRRNGIFKKAQELTVLCDAKVSLIMFSNTGKFHEYISPSITTKKIY

DEYQKALGIDLWSTHYERMQEHLKKLKEINNKLRREIGHRIGEELNDLSIEELGGLEQKMDASLT
TVRERKVVASTLSRFDCLVVTTVVVQKANGLWVDDKAIEVKHANLGKKKLGGVVPTKPGPHPA
KGQNGHRGEGRRWVPVLTDQRSYAEANQEMKELGSSNALQNQDMARTNRDKEGDADMEDDS
DHGLDI

>CchMADS09

MGRVKLQIKRIENNTNRQVTFSKRRNGLIKKAYELSVLCDIDIALIMFSPSGRLSHFSGKRRIEDALS
RFVNLADHDRGGILQNREDFQQEINNLQQQLQMAEDQLSIFEPHVLGFTSMEELESCEKNLLESM
NRVMQRKKYLMGDHLSTFDMQMFFDTEEEGMPTASFQNELVCWLPENNNGGDNQNHIFGAST
SDHSCIRLGSNSAATIYNEIPDNASAGGVNGEANSIGSGGYPSDDHNSLSHQWHHASASTHDLFS
SLIPPTSFPQTQSRSHFVNESQNTCGLGAFRDLRTRTMGVMEFEQRT

>CchMADS10

MGRGRVQLKRIENKINRQVTFSKRRSGLLKKAHEISILCDAEIALIIFSTKGKLFEYSTDSCMERILER
YERYSHAERQLIATDTESQGSWSLENAKLKARLEVLQKNQRHLMGEDLDTLNLKELQNLEHQLD
SALKHIRTRKNQLMYESISELQKKDKALQEQNNLLTKQIKEKEKKEKEQQAQLEQHQNHDLNSS
SVVISQSLHSLNIGGSGAYQAAVGDVEGAPHQIQNNAVMPPWMISHING

>CchMADS11

MGRGRVELKRIENKINRQVTFAKRRNGILKKAYELSVLCDAEVALIIFSNRGKLYEFCSSSSMMKTL
ERYHKCSYGSLEANQPTIANQNGYHEYLRLKTRVEILQQSQRNLLGEDLGPLTTKELEHLEHQLE
NSLKQIRSTKVHNNAICGNPTNKTLVSKLFCPLFMATNDDVPKSIIDDITPESVRWHDDLPSFRRW
LVWKPPCEGPLETRETNVQGVLGTH

>CchMADS12

MGRVKLKIKRLESTSNRQVTYSKRRNGILKKAKELSILCDIDLILLMFSPTGKPTLFRGERSNIEEVIA
KFAQLTPQERTKRKLESLEELSNQVRMLQAQLTEMHKRLSCWSNPDKIDNIEHLRQMEDSLRESL
NRIQIQKENFAKHPLMSLECTSQFQNGMHLPLMMGGMQEAPLSWLPDNENQHMLLTGDPNFL
PQRDVECSTDHSLPTYSGYFGPGKTEIENTRQVDNTRQEGGALHGLDSTACLRLQLNEQYSYHPY
SGLDLQEAKKLRPETEMNLHGNPLDYQINSNFQLSRPIYDNMHHAWVPPPEPCSISMFNGNSYS
QQPH

>CchMADS13

MELIRNEKARYVTYQKRTKALKKKTCELETLCDVQVCLIIYRPKLDDYSTKVEIWPQNPNVIQRLI
DSYRNQSIEDRHRRTLDLSNFFENRNQKIEDALVKLRRKNDKALYSTWDNRYNDLLEGQLRAFE
GMLEGKLADVKARIEFMKGTQALASLNDMEQTTQQNHSNYFMQGLFGTTSLQMGIIHEETPISL
LNSLYEIDDISLHYPFDQTDHQRIVALDAKSMAISPMMMPMNNINDTHYTQLGGMSSNNLQCID
PLEKSIYYDPIMLLPSPEPVLTGVSCTESSFSMSRMMENEVGKNHHPSPSMGYCSGSSMQTMSPYM
QYLKMASGPSQVHAWQMEEYYKANEFQMKNQNNQRGLLALGATDLPPSSIDTTLFTHIYYAFLS
PNNVTFKFDISNSKASMLLDFTSTLHTKKPPVKTLFSIGGVGKDLSIFSNVVSSGHSRRSFIESSIEVA
RKFGFDSVDLDWESPQNPKEMENLGLLLHEWRAKVHKESKATGQAPLLLIAVMYFSVDFFLSDV
QRS

>CchMADS14

MGRGKIVIRRIDNTTSRQVTFSKRRSGLLKKAKELAILCDAEVGLIIFSSTGKLYEFTSTSMKSVIERY
NKSREENHQLHNQVSDVKFWQREAAILRQQLHNLQENHRQLMGEELYGLSVKDLQNLENQLE
MSLQGVRMKKEQILTDEIRELNQKGNLIHQENVELYKKVNLIHQENMELYKKDQGFSLQVYGTR
DVIAVTENTQNPYGFSIGDDSCVPIHLQLSQPEHQIHEIPTRETRSR

>CchMADS15

MGRGRVQLKRIENKISRQVTFSKRRSGLLKKAHEISVLCDAEVALIVFSTKGKLSEYSTDASMEKIL
EKYERYSYAERRLASPDSELQGSWSMEYPKLTARIEVLQRNIRHYVGEDLDPLSLRELQNLEQQID
TALKRIRSRKNQLMHESISELQKKEKALHDQNYLLANKLKESEKTMAEQGQWEQQTQNSSALTL

PQPPQPPPPPTLHSLTIGGTYLQARGDDDDDAHARPSPNALMPPWMLRHMNH

>CchMADS16

MGRGRVELKRIENKINRQVTFAKRRNGLLKKAYELSVLCDAEVALIIFSNRGKLYEFCSTSNMPKT
LERYQKCSYHTPEVNHLAKEIEQSSYREYSKLKDKYEALQCYQRQLLGEDLGLLNIKELEHLEHQL
ETTLKQIRSTKTQSMLDQLYDLQTKEKLWLESNRALETKLDEICREHHIRSSWAGSEQCSIFSQQQ
QHPQSQVFFQPLECNSSLQIGYNPEVSNQINAAANHDDPNMNSFIPGWML

>CchMADS17

MGRGKVQLKRIEDKNSRQVTFSKRRTGLIKKARELSILCDVEIALIVFSARGKLYQFCTGDSLRKVL
ERYQIHKDAEVAGSSVQESKKLTEGYMDFSRGTDLLQMVQRHFEEQKIEQLDVAELTQVEHQLD
AILRQTRIKKSQLMMKAVTALHEKEEQPREGRQLMEKKITAMINEATMDDDCRRRHHQQQQTQ
QGDPDMDLELYGYTNNTNNNNNNSSTGSGGGGVYHHLQQEESMFYLL

>CchMADS18

MKPCTLFTDLAKISISGNGLPFPLLFLIPCFIALFIFIFIQIQTLIRREQEQEQEQPSFADRKMARGKIQI
KRIENSTNRQVTYSKRRNGLFKKANELTVLCDAKVSIIMVSSTGKLHEFISPSTSTKQMYDQYQKA
LGIDLWCSHYERMQEHLKKLKDVNKNLRTEIRQRMGDCLNDLSYEELCGLEQDMESSVKIIRDR
KYKVLNNQIETQKKKKRNVEEIHRNLLHQVNSKEEDPQYGLVDNGVDYNSILGFSNGGHGILAL
RLQPNHHNLHDGAGSDLTTYALLE

>CchMADS19

MGRGKIEIKRIENSSNRQVTYSKRRNGIMKKAKEITVLCDAQVSLVIFASSGKMHEYCSPSTTLVDI
LDKYHKQSGERLWDAKHENLSNELDRIKKENDSMQIELRHLKGEDITSLHHKELMAIEEALENG
LGSVREKQASLMILLMEYIDMMEKNKKTLEEENKHLNFMLHQQEMNMESSREMENGYHQRVR
DFQSQMPFAFRVQPIQPNLQERI

>CchMADS20

MENKNKGGKKSKLVRLNNSQLSFLKRRFSLFKKANELSTRCGIEVAVMLVLQRNPSIDELRLGTA
RKNEALFGASIEELSLEQLKELKAKLMEVREKVTNKLVELLKEANGGGGFEMAHGF

>CchMADS21

MAKKPSMGRQKIKIAKIEIKNHLQVTFSKRRSGLFKKASELCMLCGIEIAIIVFSPAGKVFSFGHPNV
DSIVDQFLTRNPPPNTTTCHLIEAHRNASVRELNLQLTQVLNELEVEKRHGETLDHTRKASQRQY
WWEAPIDKLGLHELEQLRNSMVELKKNVTNQANKIQFEGTTNPSPFFLVNGTRMVDHFESKTSH
INASSSITPNVHNLGYYGHGFF

>CchMADS22

MARKSKGRQKIEITTMSKESNRLVTFSKRRSGLFKKASELCTLCDAELAIIVFSPGKKAFSFGHPCV
DTIVDRFLYRNPQPNSGSLQLVEAHCNANVCELNLHLTEALDTLEAEKKKGKELNKMRKDSRDK
CWWEAPVSELGLQQLEQLKVAMEDLKKNVVKQGDKIQMEESNPSRFFAAGSSSMGGHVGGGP
HDVKVSGLGLSMTPHGYTLGYGHGFF

>CchMADS23

MGRGKIVIRRIDNSSSRQVTFSKRRNGLLKKAKELSILCDAEVGLIIFSSTGKLYDFASTSMKSVIERY
NKLKEEQRQLLNPASEVQRQLSAFSPEPDLSESRSIQETLPESIG

>CchMADS24

MGRGKTVIRRIDNSTSRQVTFSKRRNGLLKKAKELSILCDAEVGVIVISSTERFYEFASSSMRSIFERY
NKGDDEHQLLSPTSEAKFWQREAESLRQQLNYLQESHRQLMGEELSGLSVKDLENLESQLETSLK

CIRAKKDQILTDEIKEVNHQGVIIHQENMKLFKEINALQKENAELQKKVYGAKDKNDINRSFHEP
HDSSNGYDLHIPTCLQLCQPQQIDGETPEKAADLGRLQLQ

>CchMADS25

MVRQRIQIKKIDNVTARQVTFSKRRRGLFKKAHELSTLCDAEIALIVFSATGRLFEYASSSMKQVIE
RHNLQPQNLVHLNQPSLELQLENSTRAMLSKEVEERTLELRQLRGEELHELGFEELKKLEKSLEG
GLSRVLKTKDDRVDKEIATLRRKEARLMEENAWLKQQVQMQIVNMGQPQEQGQSSESITNNGS
TVAPPQDYDSSDTSLKLGLPYQS

>CchMADS26

MTRKKVKLAFITNESARKGTFKKRKKGLMKKVSELSTLCGIDACAIIYSPYESQPEVWPNTLGVQR
VLAKFKRMPEMEQSKKMVNQESFIRQRIVKANEHLKKQHKDNREKEMTEVMYQCLTGRGLQN
LVIEDLIDLGWLIDQRSTNEMRQDQKPNLDLAMEAMQRPQWFNEWMNHPNEHMGFGGDEM
MMPFADNHNAMWSSVFFP

>CchMADS27

MTRKKVKLAFITNDSARKATYKKRKKGLMKKVSELSTLCGIDTCAIIYSPYESQPEVWPNTLGVRR
VLAQFKRMPEMEQSKKMLSQESFIRQRIVKAHEHLKKQHKDNREKEMTEVMYQCLTGRGLQNL
VIADLHDLGWLIDQKLEEIDKKIESVKKKRSSDLSMVPMTSRRLENLEMADLHDLGRLIDQKLKQI
DKQIESMETQTPSGVSLALGTSKSIDEKRQDQSQI

>CchMADS28

MENKLTKGRQKIEMKLIENEHARFNTFSKRKAGIFKKASELCTTCGVDIGIIFFSPTGKPFSFAHPSI
ESICNRFTDPNQISNDITTSFVEAHCQEKKHELNQQLNGVLKQLEAAKKKEKLLDQMEKELQSKG
GWKAPIDELTIHELEQLKIQMQQLQKNVRAQLDEERNKASSSSSASALPDPSHAIDPFNDGATN

>CchMADS29

MGRGKIEIKRIENANNRQVTFSKRRAGLLKKAHELAILCDAEVAVIIFSNTGKLFEFSSAGGEEEGI
RWSTSKRRRFELKSCYSMNRTLSRYNKCLDSSQTALVEHEEEEHEEEKQEFEEVDILKDEIANLKV
KQMQLLRKDLIGLSLKELHHLEEQLNEGLLSVKEKKEQLLMEQLEQSRVQVQELRNFLPLCEHSG
MPYQEYFPAERNNSLLNHGAAGPDTVHNCAVDLQLGLSSEVSCKRKAPEKENRCSNSGSQMSLR

>CchMADS30

MTRKKVNLAYIENESARKSTFKKRKKGIMKKVNELSTLCGVDACAIIFSPYDAEPEVWPSHLGAQ
RIISRFRRMPELEQSRRMVNQESFTRQRLTKAEEQLRKQHKDNRHKEMTHVMFQCLIGEGLQYL
NMVDLHDLGVLLNQTMRDVDSRIELLTEAVLPQDMAMVNAMGRGENSFNAQMPSGMAMPT
MQNQQPPRSLLRSNVHFNAQMPSGMAMPTMQNQQPTRSLLRSNVHPGLDGNVAYTFPLQWSR
G

>CchMADS31

MGRGRVELKRIENKINRQVTFSKRRNGLLKKAYELSVLCDAEVALIIFSSRGKLYEFGSAGMTKTLE
RYQHCNFNPHDNSVERETQSWYQEVSKLRAKFESLQRTQRHLLGEDLGPLSVKELQNLEKQLEG
ALAQTRQRKTQIMVEQMEELRQKERQLGDMNKQLKIKVSLELSSLQTEGQGLGPLPCSWNPTNA
STGNTSFSVHPSQPNPMDCDNETVLQIGYQHYVAGESSAPRTMAGDIVQGWVL

>CchMADS32

MVRGKTQMRRIENATSRQVTFSKRRNGLLKKAFELSVLCDAEVALIVFSPRGKLYEFASSSMQETV
QRYQRHTKDVQSNNALLLEHNMQHLKDEAADMSKKIELLEAAKRQLLGECLGSCTIEELQQIEQ
QLERSVSTVRARKMQVFQEHIAQLKEKEKILAAENAVLCEKYGLDPQQESNEDMEIVACTNTTE
NLDVETELFIGPPDARIKRALQK

>CchMADS33

MTRKKVKLAFTTNDSARKVTFNKRKKGLMKKVSKRSTLCGIDGCAIICNPYESQHEVLPNNSGVE
RVLAQFKRMPELEQSKKMMTQESIIRQRIAKSSKLAMTRKKVKLAFTTKDSARKATFNKRKKGLI
KKFKRMPDMEQSKKMMTQESFIRQRITKANEQLKKQLKDNREKEMTEVMYQCLSGRRLQNLSM
NTIEMLGNGMHRKEEKLLGIDLAMDALKSQYESQPEIWPNNLGVQRVLAQFKRTPDMEQSKKM
TTQESLIRQRITKANEQLKKQLKDNREKEMTEVMYQCLSRRRLQNLSMVDQCDLNWLIDQNLKE
IGKQIELLKKTP

>CchMADS34

MTRKKVKLAFTTNYSSRKATFNKRKKGLMKKVSELSTLCGIDGCAIICSPYESQPEVWPNNSRVQR
VLAQFKRMLEMEQSKKMMTQESFIRQRIAKANEKFGQIFE

>CchMADS35

SPTCTGGAGGHPPLGVPSTIIWGFRVLTVRANTLLPNDSEMVRGKVQLKRIENVTNRQVTFSKRK
NGLLEKAYELSVLCDAEVAVIIFSQKGRLYDFSSSKYVSCFSIFFSQSFCLFDIYVMYGKEINLLV

>CchMADS36

MVRGKVQLKRIENVTNRQVTFSKRKNGFLKKAYELSVLCDAEVAVIIFSQKGRLYDFSSSNMQKTI
ERYGEHVKVVQTNNPNEEQFTQQLEHEATIMSQKIKIIEAYQRKFLGQDLASCSVEELGEIGSQLE
QSLRSVRARKELLFKEQINQLKEKERLLLEENAKLHEKCAEKPWQLQPAKEKEVAMSSRSSLSSEM
VETELFIGLPLMRCS

>CchMADS37

MGRGKVVLERIKNMSNRQVTFSKRKNGLAKKALELSVLCDVEVALIIFSSHGKLHEFGSSSVRQTI
QRYRRCRCTSQDKTTTEQESLYLELTKLKATCESLQHSQRQLLGQDLGSLNVKELQHLEKQIDEGL
SKARQKKTEMMLEQMEELRKKEHDLEEKSKLLKAKLEELDEQVQSLGSGSATMGNNDIQVHPS
QSNPNECEDCEVLNPDLPHWAY

>CchMADS38

MQPLWKMMVFPNQESEEGSSQRKMGRGKIEIKRIENTTNRQVTFCKRRNGLLKKAYELSVLCDA
EVALIVFSSRGRLYEYANNSVRATIERYKKACSDVPTTGSVSEANTQFYQQESTKLRRQIKDIQNSN
RHILGEALSSLTFKELKNLESRLEKAISRVRSKKNELLFAEIEHMQKREIELQNANMYLRAKIAENE
RAQQQEQEQEQMNLMPAGGGGGGGMGAYDARNFFPVNLLEPSPPPPNHHYSCHDQTPLQLV

>CchMADS39

MATNPLVSESSLLPTTASSFAVTSKNLLFVPFLPISPFMMSMGRTKFSVRKIDNPTSRQAIYLKRRDG
IIKKANELSVLCDTDVGVIIFSPSGKLTSFASNGRVEDVFLRFINRPDGLKGGPIWNEECLSRRLQQL
KQEAEMLEKIEKIEELQETLYKLNRQLLEAQEKMRYYEPNVEKITSLFEAGVYQQSLENAIQCIEQS
KAKFVGNRIVQQSNDNKEVAASFAVVDEETNTAESSNANKNRNPLRDEHHHKEGNSAGPHLSL
HFLETQKNWNLSRGGQAPRFNIK

>CchMADS40

MTRKKVKLGFITNDSARKATFKKRKKGLMKKVSELSTLCGIDACAIIYSPYESQPEVWPNNLGVQ
RVLAQFKRMPEMEQSKKMMTQESFIRQRIAKANEQLKKQLKDNWEKEMTEVLYQCFSGRGLQN
LSMVDQRDLEWLIDQNLKEIGKKFESLKKTPQQVAPVALKNTKDMLGNGMHRNEEKLLGVDLA
MDAMKRSHWFNEWMNNPHYENMGFDCHFTDYDGDRAARSARGGPGGTPWRVRGSALEAKC
KIAFKVACPPLVTRAKGAWLERSHLWKTRVFGSSLERASQSQISINGSSLRQFQGEDGVGRGTEGL
VQFGRRFAR

>CchMADS41

MVRGKTQMRRIENATSRQVTFSKRRNGLLKKAFELSVLCDAEVALIIFSPRGKLYEFASSSMQETIQ
RYQRHTKDVQTDNSSVEENMQHLRHEAADMAKQIELLEVAKRKLLGEDLGSCTMEELQQIEQQ
LERSISRVRAKKMEVFMEQIDQLKEKEKVLTAENAKLCEKYGLLPRQESNEEREGVPYTESSEISDV
ETDLFIGPPEGRVKRSSPQK

>CchMADS42

MVRGKTQMRRIENATSRQVTFSKRRNGLLKKAFELSVLCDAEVALIIFSPRGKLYEFASPSMQETIQ
RYQRHTKDVQTDNSSVEENMQHLKHEAADMAKQIELLEVVKRKLLGEDLGSCTMEELQQIEQQ
LERSVSRVRAKKMEAFMEQIDQLKEKEKVLTAENAKLCEKYGLLPRQESNEEREVVPYTESSEISD
VETDLFIGPPEGRIKHASPQK

>CchMADS43

MGRGRVQLKRIENKINRQVTFSKRRGGLLKKAHEISVLCDAEVALIVFSHKGKLFEYSTDSSMEKIL
ERYERYSYVERQLINAPQTPGNWSLECTRLRAKIELLQRNHRHYVGEDLDSLSLKELQNLENQLD
TALKHIRTRKNQLMHESISELQKKEKAIQEQNNMLEKKIKEKEKEREKTIAEQSQWEQQYHCPNS
SSFLLPQQLPCLNIGGTYQGEEQEGRRNELDLTLEPIYACDLGCFAS

>CchMADS44

MGRKKVEMKRIESKSSRQVTFSKRRGGLVKKARDLSVLCDVDVALLVFSSRGKLYQFCSANNSM
AKILKQYHHHPGAKGRFSTGDCEAESRFASFRTISELLQITKRHLEEPYVDQLSVTDLIQLEKEFDA
ALTQIKSRKTLLMMESIMALHEKERILQEENKLLANQIAAMETNGNKAENKVVMASNDHLGNK
YMNSHSQQPMLHLL

>CchMADS45

MGRGRVELKRIENKINRQVTFAKRRNGLLKKAYELSVLCDAEVALIIFSNRGKLYEFCNSSSMLKT
LERYQKCNYGAPEPNVSAREALELSSQQEYLKLKARYEALQRSQRNLLGEDLGPLNSKELESLER
QLDMSLKHIRSTRVLDLPTTQYMLDQLTDLQRKEHALNEANNTLKQRLLGGNPINSLQWNLSA
QDVGYGRQPVQPQGDTFFHPLECEPTLQIGYQNDPITAAAAAAAAGPSVNNYMTGWLPC

>CchMADS46

MENKQKKHSMGRQKIEIKKIEKKSQLQVTFSKRRVGIFKKAGELSVLCGAHVAVIVTSPAGKVFAF
GNPSVDSVIDRFLSKNPNPNPNSHGIDTCHDDELHQVHQQSINPNPNPNPNPNSQCSELHRVSK
NPNPNPNSQCLDMCHEEEVRQVQQLSKKYVKAVGKLEAEKERGKAVAELNSGGGGGGFWWD
ESVEGLELHELEQYVAALEVLKSNLLARADEMAVAASGFPANFLASNGVVVADAFGSAECSGFG
FDCKPF

>CchMADS47

MSYLNQSMEVLDSPQRKIGRGKIEIKRIENTTNRQVTFCKRRNGLLKKAYELSVLCDAEVALIVFST
RGRLYEYSNNSVKGTIERYKKACSDSSNTGSVSELNAQFYQQEAAKLRGQISNLQNSHRQMLGES
LSSMSIRDLKNLESRLERGISRIRSKKNELLFAEIELMQQREIDLHNSNQYLRAKIAENERAQQQMN
LMPGGGLYKLIYMLKGIVISPVPFPSCRSTTFTR

>CchMADS48

MESSKKQTKGRQRIDTSRRRESEEDRLITFSKRRSGLYKKASELCALCNTELVVLMFSPSGKPFSFA
HPNIDVIARRFLKLPQEEDVTGGIINARLRNRINELNERLTQLGDEAQAEKERGQMLSHMAKVRP
NKPLCDANIDELSDNDAGLLKAWLLELQDRIHNRFQELAADATTYAGAPASLVDDTNVASSRQ
GHGGMHF

>CchMADS49

MERKQTQGRKKIEMKMIADENARRITFSKCRHGLFKKASELSTLCGVDMAIVLFSMGGKAFSFGK
PNVDPVVDRFLNQNAQPSEGVSSHATRHDDATVHQLNQ

>CchMADS50

MESKQTQGRKKIEMKMIADENARRITFSKRRHGLFKKASELSTLCGVDMAIVLFSMGGKAFSFGK
PNVDSVVDRFLNQNAQPSEGVSSHATRHDDATVHQLNQQFHNLRKRLEAEKKKEKIVQNMSN
NNHDLLSCRFDAYVNQLDLQQLEQLKKSMEELKKNVAQRVDELNSEGSSKHAQEEEQVENTEI
DDNDVSTIPHDWLRL

>CchMADS51

MGRQKIKIAKIEIKNHLQVTFSKRRSGLFKKASEFCTLCGVEIAIIVFSPASKVFSFGHPNVDSIVNQF
LTRNPPPNTTTSHVIEAHCNASTMREKLAKDNVGGKLQ

>CchMADS52

MERKSKGRQKIAMTRISNKSNRMVTFSKRRSGLFKKASELCTLCGAEIAIVVFSPGKKAFSFGHPC
VDTVVDCFLSRNSPPNSGSLQLVKAHCNANVSNLNLQLTEALDLLEAEKKRGEELNKMRKASRD
GCWWEAPVSELGLQQLEQLKVAMEDLKKNVAKQGEKIQMEESNPWKFFEAGSSSMGGHVGGA
SHDVKVSGLGLSMTPHGYTLGYGHGFF

>CchMADS53

MERKSKGRQKIAMTRISNKSNRIVTFSKRRSGLFKKASELCTLCGAEIAIVVFSPGKKAFSFGHPCV
DTVVDCFLSRNSPPNSGSLQLVKAHCNANVSNLYLQLTEALDMLEAEKKRGEELNKMRKASQD
GCWWEAPVSELGLQQLEQLKVAMEDLKKNVAKQSEKIQMEESNPCRFFEAGSSSVGGHVGGAS
HDVKVSGLGLSMTPHVCL

>CchMADS54

MGPKIKDVRIRIQMERKSKGRQKIAMTRMSNESNLIVTFSKRHSGLFKKASELCTLCGAEIAIIVFSP
GKKAFSFGHPCVDTVVDCFLSRNSLPNSGSRQLVKAHCNANVSKLNLQLTEALDMLEAEKKRGE
ELNKMRKAS

>CchMADS55

MGRVKLQIKKIENTTNRQVTFSKRRNGLIKKAYELSVLCDVDVALIMFSPSGRLSIFSGNKSMEEIM
ARYMNLPEHERGRLHNQEYLQRALAKLKDHNNYQAGAASPVNADSQLEEIQQEIHRSRSQLEE
MEKRLKIFEGHPSEITTVCEAQRREHILEETLKCVRIRKQVLEEKYDYSNAQLTSQVNLPLEPVLNV
DGFVTTNPNHILDWMPQPQPQPQRDSQQVHIMNFLDSNGLLPLREQGPGIGNNMLGPPSSFLNG
QNMQLDDQTSPSSCGMEDDDNNINNLRLQQQPHFGIDTIDANISPWTTTPTQFYPTGNGSFANA
QPTERALLELFLSQLTP

>CchMADS56

MGRGKIVIRRIDNSTSRQVTFSKRRNGLLKKAKELAILCDAEVGVMVFSSTGKLYDFSTSSMKTIIER
YNKSKEEHNQLANPCSEVKFWQREAAMLRQQLQNLQGNHRQMMGEELSGLNVKELQNLENQ
LEMSLKGVRMKKDQILVDEIQELNRKGNLIHQENVELYKKVNLIREENMELYKKHLRIPQVYGTR
DVNGANRNALLSNSLSLGDDSHAPVHLQLCQPQQQNYEPPSRG

>CchMADS57

MAREKIKIKKIDNITARQVTFSKRRRGLFKKAEELAVLCDADVALIIFSATGKLFDYASSSMKDILR
KYNVHANNSEKMDQPSLELQLENTNNHARLSKEVSEKTNQLRQMRGDDLQGLNIEELQQLER
MLESGLSRVLKTKGERIMNEIATLQSKGALLIEENKQLKQRMTMISKGKWGVGVANVGAESDNL
VPEEGQSPESITNGCSCNSAPPPEDDCSDTSLKLGSQIYCGFCKFEISDEYEQSSTNSINTNWSRSGV
RVGRSTDLATNLSISSNISHLNISISFSIMVVLAKATYQLAQTVRKEMEEIPLGINAFAACS

>CchMADS58

MGRGKIEIKRIENTSNRQVTYSKRRNGILKKAKEITVLCDAKVSLVVFGSSGKMHEFCSPSTTLVDI

LEKYHQQSGKRLWDAKHENLSNELDRIKKENDSLQIELRHLKGEDITSLQPRDLMAIENALETGL
ESVRYKQSEIHRMMKKNGKMLEEDNKQLNLILHQQEMEKGSREFENGYHRQVNDYQPQMPFTF
RVQPIQPNLHERI

>CchMADS59

MVAWQKQWLLNVNSNNLFMLSSKGPSRTTSFLQFTLVPQHSQVMEIHSYAGRTHERKPSENRRG
TRSKTGDIMGRGKIEVKRIENNTSRQVTFSKRRTGLLKKTHELSVLCDAQIGLIVFSSKGKLFEYCT
HPLSMGEMIGRYLDATGIRIPEHDDRLQEQIFNELTRIRNETHNLQLSLQRYKVNKVRARKFQLLQ
QQLDNLQRTEKMLEKENQDMYQWLMSNHMKQQAEELDHHQQAMTELRLVGQQHQQQVLE
QFPFYGEEQPSSVLQLASVPLQLHHHHPYQLQPTQPNLQDSNLLQQTYEQ

>CchMADS60

MSLSGSAQARACKMGRGKIEIKRIENNTNRQVTFCKRRNGLLKKAYELSVLCEAEVALIVFSSRGR
VYEYANNNIKSTIERYKKAVADNSNPCPTPEINAQFYQQESKKLRQQIQMIQNTNRSLMGEGLDC
LNMKELKQLENRLERGITRIRSKKHEMILAETENLQKREMELENENAFLRAKIAETERIQEQNMV
PGEEYNAIQAYFARNDLVLQLNIMDAEHPPPPPPAAYQRFDKKSLLHLG

>CchMADS61

MTNRVKLAFITDNAARKTTLRKRRAGMIKKISELSTLCDIEAGIVIYNPGEVTPTVWPSYEYMKQM
FKRFLSMSIIDRSQKMVTYEGYIIQIITKETENNITEKKNNDKKEIQEIMNQTFEGNDLNELDMTRL
NLLSLLADDKLKQLKKKQNWPCGQQMALLPPPPLSPVLAPEIIEVEEGITAPTNGEDGESSIPMIIQ
ELMNDQCFMETTAEHMELLLSKDVEGTSSSKNEEDLPKDLNEVFPHIYFP

>CchMADS62

MGRGRVELKRIENKINRQVTFAKRRNGLLKKAYELSVLCDAEVALIIFSNRGKLYEFCSSSSMLKTL
ERYQKCNYGAPEPNVSTREALELSSQQEYLKLKARYEALQRSQRNLLGEDLGPLNSKELETLERQL
DMSLKQIRSTRTQYMLDQLTDLQRKEHALSEANKTLKQRLVEGNQVISLQWNPSAQDVGYSRQ
QAQPQGDVFFHPLDCEPTLQIGYQNDPITVAAAGPSVNNYMPGWLP

>CchMADS63

MNPTPGSETTRHSQTASVYSSQDSCNFHFPEREKGLGFVFCGCRLSRAEMGRRRVELRPILDKNKR
QITFSKRRQGLMKKAQELSVLCDVDVALAVFTSGCRAYKFSGGNRYALALSFSLFNIQILNV

>CchMADS64

MGRGKIEIKKIENVNSRQVTFSKRKAGLLKKAKELSILCDAEVGLIIFNNSGKLYEFASPRYNNGLE
SSTLTNPSTEKAVLELEKQPPELDALRGEVAKLQKGYRRTMGKDLEGMSFKELQQLEHQLNEGIL
SVKDRKEQVLLEQLEKSKLQEQKVKLENETLREQIEELGHRSTTPTPTHEFHCLGRKRSVLSSNTV
CDGYFDTERDSETSLRLGLSSEVFHKRKVCKIESVSNDDSGSQMAS

>CchMADS65

MGRRRVEIKRIEDKARRQTTFTKRRDGLFKKTGELCNLCGTEAAVITFSNAGNTFAFGNPSVDSVL
DRYLSMTSSSSRASVDGGEVAAAERVREREEKLAEALARLEVEKKRGEAIDEALSMLKLNEDVVG
DSKVRVSEEMGEIRSAVEMAQLEAEKKHDEVVDEMLAMLNAKAEASRLKEVEQEVGKDVDEET
TELQIGNKSQRGNR

>CchMADS66

MAKEDDEYSVCFSSFIVRTFRTEQSEFVWTFITEQIHSFALFGYMMSNKVKLFGSESSNNNNEKQW
EEKSSSIVLAMAKKPSMGHQKIKIAKIEIKNHLQVTFSKRRSGLFKKASELCTLCAIEIAIIVFSPAGK
AHRNASVRELNLQLTQVLNELEAEKRRGETLDHRRKARQRQYWWEAPIDKLGLHELEQLRNSM
VELKKNVTNQVNKIQVEGTTNPSPFFLVNGTRMVDHFESKTSHINASSSTTPNVHNFGYYAHGFF

>CchMADS67

MERKGKGRQKITMTKMENESNLQVTFSKRRSGHFKKASELSILCGAEVGIIVFSHGKNAYSFGHPT
VDMIINRFLSRDAPLNSPSGRGEAARHSSRSAQDGCGSRRTLLVGGSG

>CchMADS68

MERKSKGHQKITMTKMENESNLEVTFSKRRSGLFKKASELSIQCGAEVGIIVFSPGKKAYSFGQTTV
DMIIDRFLSRDVPLNSGAHQLDLNLQLTHVQALLGVEKQRGTALDQLRMAAGQGEHHWWEGQ
AEEMNLG

>CchMADS69

MVRGKTQMKRIENATSRQVTFSKRRSGLLKKAFELSVLCDAEVALIIFSPKGRLYEFSNSSISKTIER
YQRNTKEQEGSGRKEAEESLQHLKEEAVSMSQKIELIEASKRKLLGDCLESCSINELQLIEEQLEKSL
HNIRARKTLLYKEKIEQLKEEEKILTEENQKLREKCEMRSPHLPIPLLVAPEPNREFSDVETELFIGLP
EQRTTHSL

>CchMADS70

MDSYWHCYHFSIVKSCFVRTMKITCMYQKEQHIGVTVSMVRGKTQMKRIEKATSRQVTLSKHRS
GLLKKAFELSVLCDAEVALIIFSPKGRLYKFSNSRAAAMGDWDLSLFGVVSCFWTSFSSWSFFLSGL
VPHQTPGVEFADDWALFSVPT

>CchMADS71

MARGKVQMKRIENPVHRQVTFCKRRAGLLKKAKELSVLCDAEIGVLIFSANGKLYELATKGTMQ
GLVGRYMKSTGDTQADHDEEKQVMDSKEEINMLKNEIEFLKKGLRFMSGGGVEAMTLDELHML
EKHLEIWIYHIRSVKMDIMSQEIQLLKTKEGILKAANNYLQEKIDEQYGTTNYGEVITNPHPLTIQN
EIYQF

>CchMADS72

MLPLIIIPISISPTFSLHFSIFRLLFSLHKPSFDSTFVDFVFKGFALNFLGKMVRQRIQIKKIDNVTSRQV
TFSKRRKGLFKKAQELSTLCDAEIALIVFSATGKLFEFSSSSMRQVIERHNLESGNLVNLNQPSLEQ
QLENSGCTILSKEVNKKIHELRQLRGEELQGLDAEELKNLEKSLEGGLSRVLMTKGEIMEKEITAR
KTKEARLVEENVWLKQKVPMEIGQTHDDQQGQSAEFITNNGSSAASPQDNGSSDTSLKLGLPFPE
LNLRARMINA

>CchMADS73

MGRGKIVIRRIDNLTSRQVTFSKRRNGLLKKAKELSILCDAEVGVIVISSTGRLYEFASPSMRSIFERY
NNEQENHQLLSPTSEAKVFFHEQLAYYLASFPALVQDIQLGLSHLLFNFLKFLALAKGGRKLKATI
ELLTRKPQIQSPELHLHFHCDNNFRKLTGEELSDLSVEDLENLEGQLETSLKGIRTKKHQILTDEIQ
ELNHQGVISHQENMKLYTEVKVLRRENAELHKKVYGANDINRSSFKPYGCSNGYDLHVPIQLQL
SQPQQTHNETPETQGI

>CchMADS74

MVTMNTTKKTTQGRQKIEIKKIESVSKRQVTFSKRRAGLFKKASELCILSGAEIAIVVKSPGKRTFAF
GHPSVDAVIDRYLTGTSAAAEQNNRPSVLDFNDKYAQVSKELEAEKRRSAVIEETKKAANDGGF
WWDEAVDDLGLEELEQYVAALEELKKNVSMKADELMIMKANSSMMFGMNESANHGGLGLMS
DCATSMVPHGFDFANSSMMFGMNESKNHGGLGLMSDCAASMIPHGFDFGRGQF

>CchMADS75

MERKSKGRPKIVMTRMSKESNLLVTFSTRRSGLFKKASELCTLCGAEIAIVVFSPGKKAFSFGHPCV
DTVVDCFLSRNSPPNSGSLQLVKAHCNANVSKLNLQLTEALDMLEAEEKRGEELNKMRKASGD
RCWWEAPVSELGLQQLEQLKVAMEDLKKNVAKQSEKIQMEELNPSRFFVAGSSSMGGHVGGAS

HDVKVSGLGLSMTPHGYTLRYGRGFF

>CchMADS76

MERKQTQGRKKIEMKMIANENARRITFSKRRHGIFNKASELSTLFGVDMAIVLFSIGGKAFSFGKP
NVDSVVDRFLNQNAQPSDGVSSHATQHDDATVHQLNQQFHELSKRLKAEKKKEKIVQNMSSN
NYGLLSCRFDAYVNQVDLQQLKQLKRSMVELKKNMAQRVDELNSEGSSKHAQEEEQVENTEIN
DNDVSTIPHDWLRLAVVQWCGDDERRTASRLAVVRRRRQMMDKSWIDLPNRMSSEYINGIDEFL
VFAYTNKVEGLMISCSCRKCENQYHLAREGVREHLILNGFFKKYKLWINHGESYVSLHDNRENET
LVNLDIGDDMIGMIQGAMGNPHVGTSVEGNKPQGPDPHASEPQRLQSISNFFKMHKVLNQWTD
KSVDMLLELLNEAFLLGVKLPDLYYKAQKVTTDLGFTYETWNACPNRCMLFRNEDANLDKCVI
CNSSRWKSNGDGGSNGPNKGGKRIAAKQMRYFPLQPRLQRLFMSSKTAKLMRWHAEEQIDDG
VFRHPADSLAWKDFDRRNIDFSNDCRSVRLGLASDGFNPFRTMNIVHSTWPVVLIPYNLPLWMC
MKQPFLILSVLIDGPKGPGDKIDVYMQPLIEELKKLWNEGWSTKGEYACPCCNKETTSCWLKHSR
KQSYLGHRRFLPTGHKFRKDKVSFDGSREVKLKPKLLSGTEMLLQLESEGILTQYKRDALKERHRR
QVEKEKAGHRTHSWKKKSIFYELSYWEHNLIRHNLDVMHIEKNVCDNVLWTILGVAGKSKDGV
NARRDLQDMNIRKPLHLQLRGSNKAYLPPAQFTMTKDEKDLFLKVLKEVRVPDGYSSNISRRGRS
HDRSIGGLKSHDSHIIMQQLIPLAIRRSLLKNVIEPLIELSNFFRQLCSKVNCATDLQYIQDRIAVTLC
HLEKIFPLSFFDIMEHLPIHLADEALLGGAIQFRWMYPIERYLLTLKQYVRNRAHPEGSIAKGYLM
EECMNFCAQYLNDVETKSTQPVRNYSGVDNLGPAIGENTRFYLDNTSWVQARWYILFNTSAVQP
FINKHLEVLKLKMPRVDDHQFRREHFETFHIWFKEHVQTLRRTTDVIFSEEIILLANGPHNLARRF
NGYILNGFQYRVKAMDDRRAIQNCGVALKADTISYASQKDKNPRVGSVYYYGQLTDIVEIRYSNE
KKHVLFKCDWIDNIHGKKKDVFNSHPLQPDWQVVVKMSQRGLFDMSSTDPQIEPSTSQQLEENI
MLHDEEVGWVREAAEGETVDIIMGRKMKPDHPRRMMSDVLHNRTSVAPPSKEASHRPPSDQPS
QQPLLLAPRLEEASQRPPSEGPLVPPHRPPSVAHSSYHVLSLHAYHRPFDFFIAHLRYKNSICHVSS
SSTLTRRGRGCAKGIREWGTGKKLDIQFNENYCPIGDNVAKLTTQLGIIVRNGNIVPLTFLDWNN
VPDDIVDVIWKDVKDNLNICPEEYKPICMKNCNSIWKDHKNKIKAKYFKPRSSDPNLKDDVPLHI
VPRQWEELVEYWRTSDAEKIATRNAKNWRAHDIAHTTGRTTFA

>CchMADS77

MTRNKIKLAWIVDKSSRKATLKRRRASMFKKAGELSVLCDVEVGVIVYSHEEADLAVWPSYGHM
KQMFERFLSIPIVERNQKMMTNEGFLTQRVTQETNKNNMEKKKNDKKEIQEIMNQIFEGSNLYQ
LNIMRLNYLSLLTVDKLKQLEERERARGQQAPPLLPLLIPAPALGPAPGMMEVNEVSQAHTSIEA
TSIEQLMNDNWFMETMPLNWDMLGPSGVSDMGMPSAPVVGRMNGDNEEDLPDDLNAFFPHIY
SP

>CchMADS78

MTGNKIKLEWIVNDSSRKSTLKRRRASIFKKAGDLSTLCDVEIGVLVYSQDEDDLAVWPSYGHMK
QMFERFLSIPIVERSEKMTTYEGLLTQRVTQEAHKNSDKLMQLEKRERARRQHALPLSPALAPAPR
PRMIAVDEAFQAHTSTEATCIEQLMNDNWFIETMALNQDMPGPSGVSDMGMPSGSVVDRMNG
DNEEDLPEDLNAFFPHIYSP

>CchMADS79

MSGDTSVDATENERFASPVANSSRVHPFKAVEFCYRKRETKEKMGSGKKKIEIKRVEKEGQRMVT
FSKRRQGLFNKARQLRSLTGADIAILTFSPAGRPYTHGEPSFDALVDRYLNTAAGEKAEEGCEAT
ATNHHRLSSWLDALQFDASDSIEDLEILKKGLEEIAAKVAEKVDDLFVDSLLF

>CchMADS80

MVRGKTQMKRIENATSRQVTFSKRRSGLLKKAFELSVLCDAEVALIIFSTKGKLYEFSNSSINKTIDR
YQTNAKDLGSSNNAAQENLQHLKEEAVSLSEKIELLQVSKRKLLGDGLESSCIDELQQVQDQIEQ
SLSKIRARKTQVFKEEIEQLKEEERILTEENAKLREKLKCDQMELPCLAFAQEVPTQREISDVETELF
IGPPDEQRTTRWLIQSSTAINGMRL

>CchMADS81

MGSGKKKIEIKRVEKEGQRMVTFSKRRHGLFNKARQLRSLTGADIAIRTFSLAGRPYTHGEPSFDA

LVDRYLNTAAAVEKAEEGCEAAAANHHYCLILIFLICQDRFELRRRRRRRRRIAFRFSGAVSRWKRSSEMIGESFDDEIHGGK

>CchMADS82

MGRGRVDLKRIENKINRQVTFAKRRNGLFKKAYELSVLCDAEVALIVFSNRGKLYEFCSGSSMAKTLERYQRCSHDLLDPRQPAIENQNNYHEYLRLKARVEILQQSQRNLLGEDLGPLNTKGA

>CchMADS83

MGRVKLKIKRLESTSNRQVTYSKRRNGILKKAKELSILCDIDIVLLMFSPTGKATLFRGERSNIEEVITKFAQMTPQERTKRSVKICYIYEDEELFFFVFFV

>CchMADS84

MRRGKVELKRIENPSSRQVTFSKRRNGLLKKAFELSILCDAEVALLIFSPSGKAYQFCSHDMDRTISRYRSEVRLSQFNDQGLRTMEALCRRRSINVEHERTEANRAPVEEWS

>CchMADS85

MAKKPSMGRQKIKIAKIEIKNRLQVTFSKRRSGLFKTASELCTLCGVEMAIIVFSPTGKVFSFGHPNVDSIVDQFLTRNPPPDTTTRHLIEAHRDASVHKLNLQLTQVLNELEVKKRHGETFDHTRKASERQYWWEAPIDKLGLHELEQLRNSMVELKKNVTNQANKIQVGGNTNPLPFFLVNGSGIVAPFESKTSHINASSSTSPNVHNFGYYGHGFF

>CchMADS86

MGSGKKKIEIKRVEKERQRMVTFSKRRQGLFNKARQLRSLTGADIAILTFSPASRPYTHGEPSFDALVDRYLNTAAAGEKAEEGCEAAAANHHRLSSWLDALQFDASDSIEDLEILKKGLEEIAAKVAEKIDDVFVDSLLV

>CchMADS87

MSGDTSVDATENERFASPVANSRCKIFILPGRAIRHQPLGRNLVQRSPNENLCWLNPFKAVEFCSRKSETVEFCYRKSETEEKMGSGKKKIEIKRVEKEGQRMVTFSKRRHGLFNKARQLRSLTGADIAILTFSPAGRPYTHGEPSFDALVDRYLNTAAAGEKAEEGCEAAAANHHRLSSWLDALQFDASDSIEDLEILKKGLEEIAAKVAEKIDDLFVDSLLYGETSPDALQYYYSDSDNLVEEAAGGGSAGKSFRERDKEGFSESEYATLVNGEILKKVVSGACVDGATATAAGSKSMRYMIQTPIDCVSAIYTVLSRRRGHVTADVPQPGSLYCQGLAFLPVIESFGFETDLRYHTQGQAFCLSVFDHWAIVPGDPLDKSIVLLEPAPIQHLAREFMVKTKRRKGMSKDVSINKFFDETMVVELSQQATDLHQQMI

>CchMADS88

MSGDTSVDATENERFASPVANSRCKIFILPVEFCYRKRETEEKMGSGKKKIEIKRVEKEGQRMVTFSKRRQGLFNKARQLRSLTGADIAILTFSPAGRPYTHGEPSFDALVDRYLNTAAAGEKAEEGCEAAAANHHRLSSWLDALQFDASDSIEDLEILKKGLEEIAAKVAEKVDDLFVDSLLF

>CchMADS89

MGSGKSKIEIKRVEKEGQRMVTFSKRRRGLFNKARQLRSLTGADIAILTFSPAGRPYTHGEPSFDALVDRYLNTAATGEKAEEGCEAASANHHRLSSRLDALQFDASDSIEDLEILKKGLEEIAAKVAEKIDDVFVDSLLL
